# Inhibitory effect of SLIT2 on granulosa cell proliferation mediated by the CDC42-PAKs-ERK1/2 MAPK pathway in the prehierarchical follicles of the chicken ovary

**DOI:** 10.1038/s41598-018-27601-z

**Published:** 2018-06-15

**Authors:** Rifu Xu, Ning Qin, Xiaoxing Xu, Xue Sun, Xiaoxia Chen, Jinghua Zhao

**Affiliations:** 1 Department of Animal Genetics, Breeding and Reproduction, College of Animal Science and Technology, Jilin Agricultural University, Changchun, 130118 People’s Republic of China; 2 Key Laboratory of Animal Production and Product Quality Safety of the Ministry of Education, Changchun, 130118 People’s Republic of China

## Abstract

The SLIT2 ligand and ROBO receptors of the SLIT/ROBO pathway are expressed in hen ovarian follicles and have been shown to play critical roles in ovary development, cell proliferation and apoptosis in mammals. However, the exact roles of SLIT2 and the molecular mechanisms of chicken follicle development remain poorly understood. Here, we discovered that high levels of SLIT2 suppress *FSHR*, *GDF9*, *STAR* and *CYP11A1* mRNA and protein expression in granulosa cells (GCs) and cell proliferation (p < 0.01). However, these inhibitory effects can be abolished by the siRNA-mediated knockdown of the ROBO1 and ROBO2 receptors. Furthermore, the activity of CDC42, which is a key Rho GTPase in the SLIT/ROBO pathway, is regulated by the ligand SLIT2 because the intrinsic GTPase activation activity of CDC42 is activated or repressed by regulating SRGAP1 expression (p < 0.01). The effects of the SLIT2 overexpression on GC proliferation and phosphorylation of the B-RAF, RAF1 and ERK1/2 kinases were completely abrogated by knocking down endogenous PAK1 and partially abrogated by the knockdown of PAK2 and PAK3 in the GCs. Collectively, our findings indicate that SLIT2 suppresses GC proliferation, differentiation and follicle selection mainly by a mechanism involving ROBO1 and ROBO2 and that this suppression is mediated by the CDC42-PAKs-ERK1/2 MAPK signaling cascade in the prehierarchical follicles of the chicken ovary.

## Introduction

In chicken ovary, the cellular and molecular mechanisms by which the prehierarchical follicles (6–8 mm in diameter) are selected to enter the preovulatory stage in which the selected follicles undergo a complicated hierarchal regulation targeting granulosa cell (GC) proliferation, differentiation and oocyte growth and maturation are well known^1,2^. A variety of endocrine, paracrine, and autocrine factors that play a pivotal role during ovarian follicle development exhibit a remarkably distinct expression pattern before and after follicle selection to coordinate the control of follicle growth^3,4^. Follicle stimulating hormone (FSH) is generally acknowledged to be required for ovarian follicle maturation, and a relatively higher mRNA expression level of FSH receptor (*FSHR*) was observed in the GC layer of the selected prehierarchical follicles^5^. Several members of the transforming growth factor β (TGFβ) family, including growth differentiation factor-9 (GDF9), and ovarian local hormones, including cytochrome P450 cholesterol side-chain cleavage (P450_SCC_) enzyme (CYP11A1) and steroidogenic acute regulatory protein (StAR), have been demonstrated to serve as biomarkers of the growth and differentiation of granulosa cells and theca cells in chicken hierarchal follicles^4,6^. As previously reported, prior to follicle selection (9–12 mm), the granulosa layer remains in an undifferentiated state as shown by the nondetectable to very low mRNA levels of *CYP11A1* and *STAR*^7,8^; however, immediately after selection, the *CYP11A1* and *STAR* mRNA levels dramatically increase in the granulosa cell layer during the investigated developmental stages^6^. In addition, the *GDF9* mRNA expression levels are decreased during this stage and accompanied by higher levels of *FSHR* mRNA expression^4^. During this key point of follicle selection, the mitogen-activated protein kinase (MAPK)/extracellular signal-regulated kinase (ERK) signaling pathway involved in the regulation of follicle growth^9^, and the SLIT/ROBO signaling and small Rho GTPase-dependent pathway has been recently identified to play an indispensable role in ovarian follicle development^10–13^. Furthermore, when activated, several key members of the Rho GTPase family (small G protein family), such as CDC42 and Rac1, may function by directly responding to the SLIT/ROBO signals and simultaneously induce the activation of targeted MAPK/ERK cascade elements^14^. However, the precise components of the Rho GTPase pathway and MAPK/ERK signaling cascade that are implicated in the regulation of granulosa cell proliferation and differentiation and the fine-tuning mechanisms of Rho GTPase-mediated SLIT/ROBO signaling in undifferentiated follicles remain largely unknown.

The SLIT/ROBO pathway comprises three secreted SLIT glycoproteins (SLIT1, 2, and 3) and four transmembrane Roundabout (ROBO) receptors (ROBO1, 2, 3, and 4) in chicken^11^. Although these components were originally identified in the nervous system as important guidance molecules to prevent axons from migrating to inappropriate locations during the assembly of the nervous system^15^, most SLITs and ROBOs have also been shown to regulate numerous processes, including cell proliferation, apoptosis, adhesion and migration, in ovarian follicles^10^. Our recent studies investigating the chicken ovary revealed that both *SLIT* and *ROBO* transcripts and their proteins are predominantly expressed in oocytes and granulosa cells in various-sized prehierarchical follicles, and the expression levels of the SLIT and ROBO members in the cultured follicles (4 to 8 mm in diameter) are hormonally regulated by activin A and/or inhibin A^11^, suggesting that the SLIT/ROBO pathway may be important for prehierarchical follicle development. Although Slit2 was found to interact with Robo1 to mediate repulsive cues in myogenesis^16^, the intracellular domain of Robo interacts with a novel family of Rho GTPase activating proteins (GAPs), and two Slit-Robo GAPs (srGAPs) are expressed in regions responsive to Slit^17^, the interactive relationships between any SLIT ligand and its corresponding ROBO receptors that mediate the regulation of ovarian follicle growth by extracellular stimuli that induce intracellular responses have not been characterized to date.

Compelling evidence suggests that the members of the Rho family of small GTPases, particularly Cdc42, play a critical role in signaling between guidance receptors and the cytoskeleton^18^. In vertebrates, the stimulation of Robo1 by Slit has been shown to result in the recruitment and activation of members of the srGAP family of Rho GAPs, which appear to have some specificity for Cdc42^17^. The activity of the Cdc42 protein is negatively regulated by srGAP1, and Slit increases the srGAP1-Robo1 interaction and inactivates Cdc42^17,19^. Furthermore, signaling from the small GTP-binding protein Cdc42 or related GTPase Rac1, which is another member of the Rho family, to MAPK pathways plays a key role in the regulation of cell growth, survival and proliferation in a variety of cell types^20,21^. Moreover, in human neutrophils, Cdc42 and Rac activate the effector p21-activated kinase 1 (PAK1) by specifically binding the p21-binding domain (PBD) in a GTP-dependent manner^22^, which inhibits both the intrinsic and GAP-enhanced GTPase activity of Cdc42 and Rac^23^. Therefore, the p21 ras-related protein CDC42 and Rac1 likely activate a downstream serine/threonine protein kinase in the MAPK/ERK pathway, such as B-RAF or RAF1, by activating the kinase PAK1 as previously reported in mammalian cells^21,24^. Moreover, previous work has shown that MAPK/ERK activation maintains hen granulosa cells in an undifferentiated state^25,26^. Thus, the extreme up- or down-regulation of the activity of CDC42 and Rac1 by stimulating or silencing SLIT/ROBO signals could be predicted to efficiently alter the levels of GC proliferation and differentiation in the follicles. Nevertheless, how do ROBOs regulate the actions of the MAPK/ERK pathway by inducing srGAP1 and Cdc42 activity? The potential role and regulatory mechanism of each member of the SLIT/ROBO family that is mediated by the ERK pathway to control GC proliferation and differentiation in chicken ovarian follicles remain poorly understood.

In the current study, to further explore the function and regulatory mechanism of the SLIT/ROBO signaling pathway in ovarian follicle development, we aimed to investigate the exact role of SLIT2 in the proliferation and differentiation of granulosa cells during prehierarchical follicle development, identify the direct physical interaction between the ligand SLIT2 and its receptors in the SLIT/ROBO pathway and effects on its potential downstream kinases, including CDC42, PAKs and RAFs, and further probe the molecular mechanism of the SLIT2 protein in the regulation of prehierarchical follicular development by the p21-activated serine/threonine kinases (PAKs) and ERK1/2 MAPK signaling cascade in the hen ovary.

## Results

### Effects of *SLIT*2 expression on the mRNA and protein levels of *ROBO1* and *ROBO2* in granulosa cells

**Figure 1:**
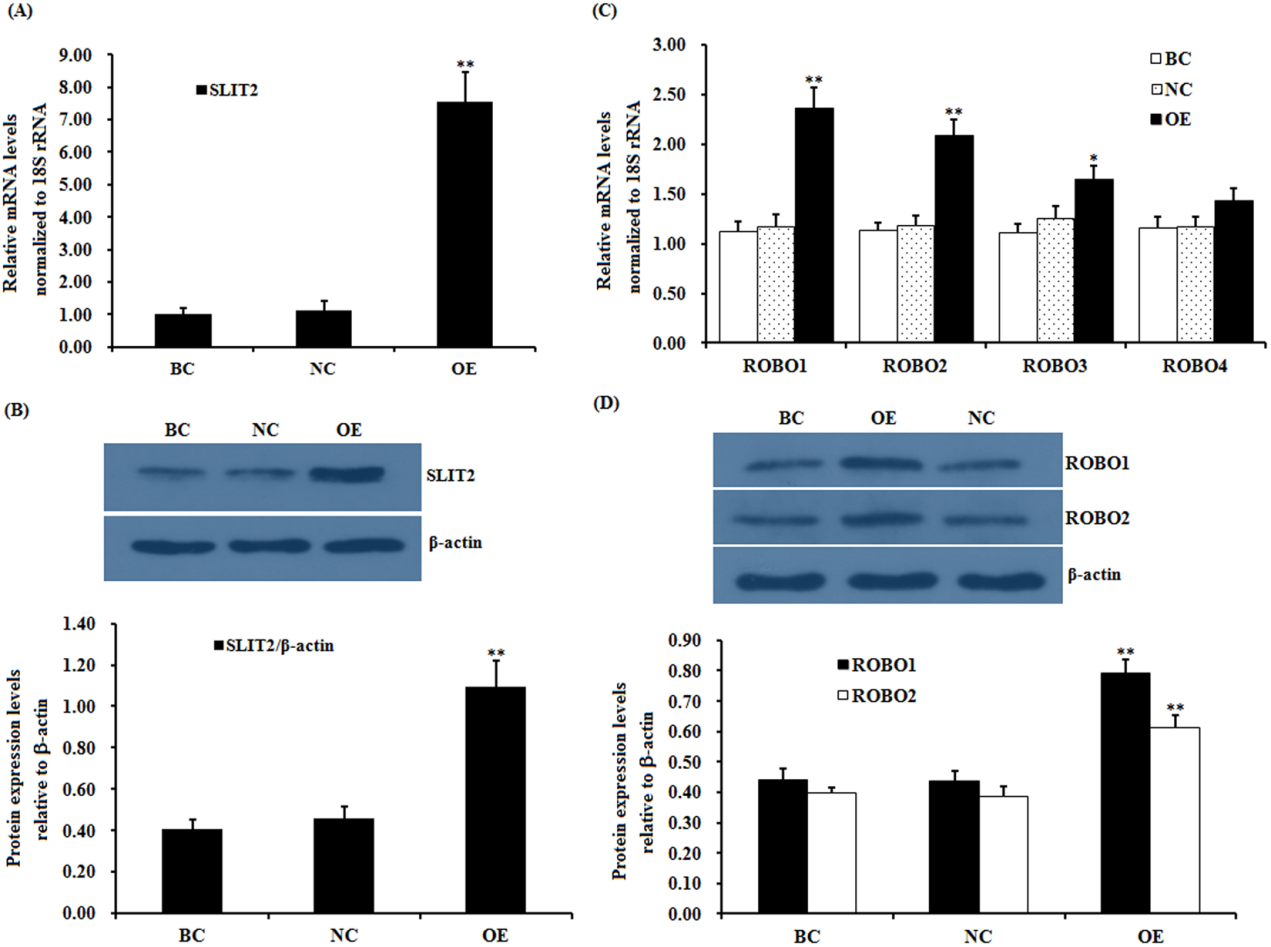
Effects of overexpressing *SLIT2* on the expression of the *ROBO1, ROBO2*, ROBO3 and *ROBO4* genes. The granulosa cells were transfected with a reconstructed pYr-adshuttle-4-SLIT2 vector, a pYr-adshuttle-4 empty vector (negative control) or no plasmid (blank control). (**A**) The expression of the *SLIT2* gene before and after the GCs were transfected with the pYr-adshuttle-4-SLIT2 expression vector for 24 h was examined by qRT-PCR. The mRNA expression was normalized to that of the *18S rRNA* gene; the values on the bar graphs represent the mean ± SEM of 10 hens (n = 10) from a representative experiment. (**B**) The expression levels of the SLIT2 protein in the GCs before and after the transfection with the pYr-adshuttle-4-SLIT2 vector were detected by western blotting. β-actin was used as a loading control. The blots were cropped, and the gels were run under the same experimental conditions. (**C**) The influence of the *SLIT2* overexpression on the *ROBO1*, *ROBO2, ROBO3* and *ROBO4* mRNA abundance in the granulosa cells from the prehierarchical follicles (6 to 8 mm in diameter) was examined. (**D**) The effects of the *SLIT2* overexpression on the protein levels of ROBO1 and ROBO2. For each group, the superscript symbol above the bar indicates that the difference was significant compared to the control group **P < 0.01, *P < 0.05.

To determine whether SLIT2 plays a role in controlling the mRNA and protein expression of *ROBO* in granulosa cells (GCs) and regulating the proliferation and differentiation of prehierarchical follicles (6-8 mm in diameter) in GCs, a reconstructed pYr-adshuttle-4-SLIT2 vector and SLIT2-specific siRNA were prepared and transfected into cultured GCs. As shown in Fig. 1, the mRNA and protein expression levels of *SLIT2* were markedly enhanced in the cells after 24 h of transfection with the pYr-adshuttle-4-SLIT2 vector (p < 0.01) as detected by RT-PCR and a western blot analysis. Endogenously expressed SLIT2 was also detectable in the cells with or without the transfection with the pYr-adshuttle-4 vector (Fig. 1A,B). After the stimulation by the overexpressed SLIT2, the mRNA and protein expression levels of *ROBO1* and *ROBO2* were highly significantly increased (p < 0.01) (Fig. 1C,D), but no changes were detected in the *ROBO3* and *ROBO4* mRNA expression levels before and after the SLIT2 overexpression (P > 0.05). These results reveal that of the four receptors (ROBO1-4) in chicken ovarian GCs, the ligand SLIT2 may mainly target and interact with ROBO1 and ROBO2.

**Figure 2:**
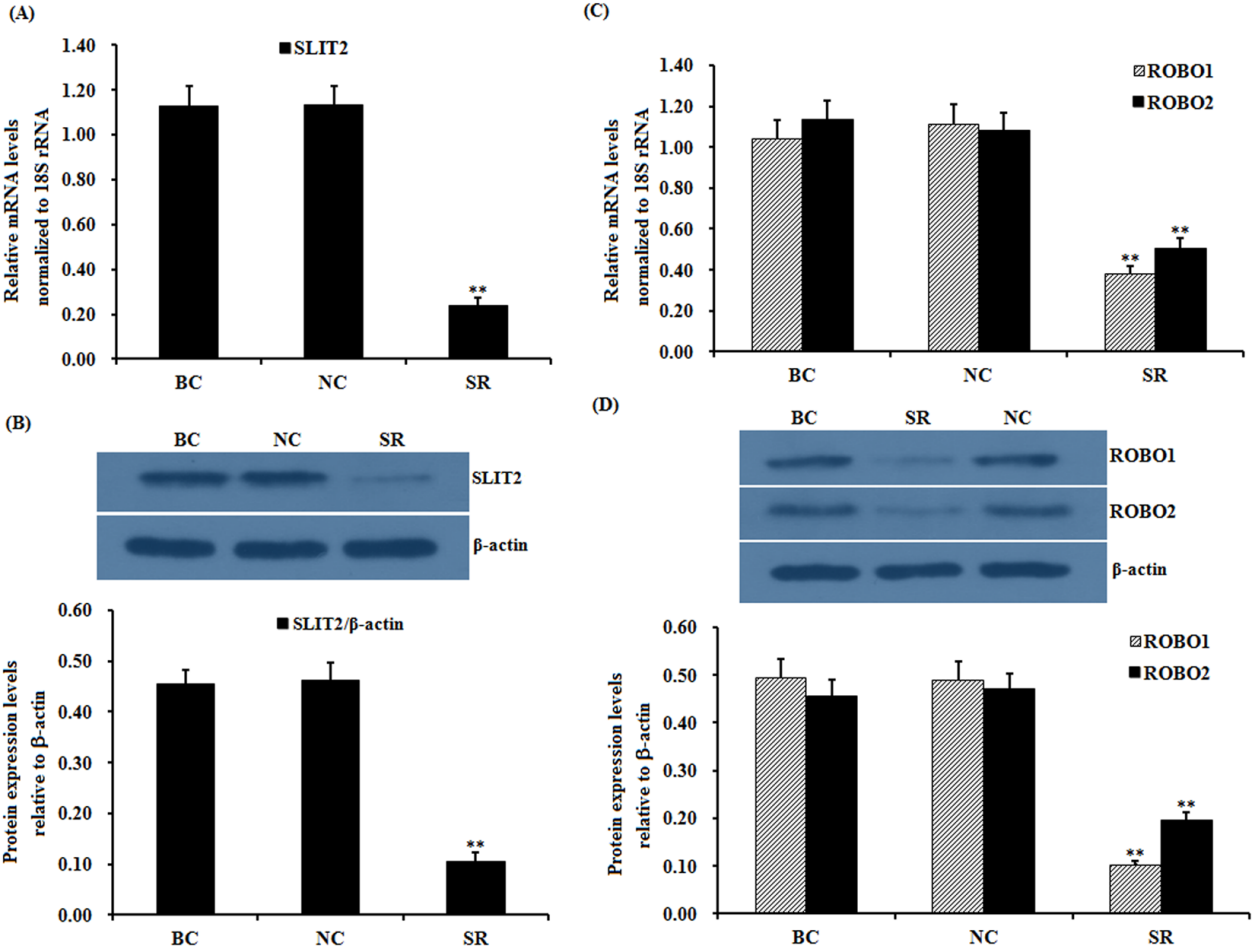
Effects of the *SLIT2* knockdown on the expression of the *ROBO1* and *ROBO2* genes. The granulosa cells were transfected with the *SLIT2*-specific siRNA, scrambled siRNA (negative control) or no siRNA (blank control). (**A**) The expression of the *SLIT2* gene in the GCs with or without the interference of the specific siRNA was determined by qRT-PCR. The mRNA expression was normalized to that of the *18S rRNA* gene; the values of the bar graphs represent the mean ± SEM of 10 hens (n = 10) from a representative experiment. (**B**) The expression levels of the SLIT2 protein in the GCs with or without the siRNA interference were detected by western blotting. β-actin was used as a loading control. (**C**) The influence of the *SLIT2* knockdown on the *ROBO1* and *ROBO2* mRNA abundance in the granulosa cells. (**D**) The effects of the *SLIT2* knockdown on the protein levels of ROBO1 and ROBO2. All blots were cropped, and the gels were run under the same experimental conditions. For each group, the superscript symbol above the bar indicates that the difference was significant compared to the control group **P < 0.01, *P < 0.05.

To further explore the potential regulation of the function of both receptors by SLIT2, SLIT2 expression was efficiently knocked down in cells transfected with a SLIT2-specific siRNA as shown by RT-PCR and a western blot analysis. The mRNA and protein expression levels of *SLIT2* were significantly decreased in the SLIT2-siRNA-transfected cells (p < 0.01) (Fig. 2A,B). Similarly, a remarkable decrease in *ROBO1* and *ROBO2* mRNA and protein expression was observed (p < 0.01) (Fig. 2C,D).

### SLIT2 interacts with the ROBO1 and ROBO2 receptors in GCs as determined by coimmunoprecipitation

**Figure 3:**
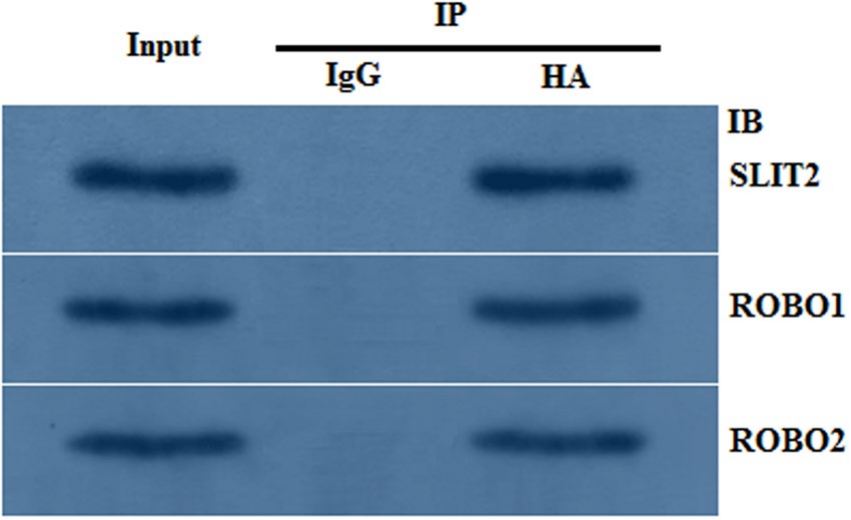
Interaction between SLIT2 and ROBO1 and ROBO2 as determined by the coimmunoprecipitation experiment. The chicken GCs were transfected with the pYr-adshuttle-4-SLIT2 expression construct. After transfection for 24 h, the cells were lysed, and the lysates were immunoprecipitated with a control chicken IgG or an antibody against HA. The immunoprecipitation (IP) with the HA antibody was analyzed by immunoblotting (IB) using the SLIT2, ROBO1 and ROBO2 antibodies. After the lysates were immunoprecipitated with the chicken IgG, no pYr-adshuttle-4-SLIT2 band was observed in the immunoprecipitants from the cells expressing HA-SLIT2, and no ROBO1 and ROBO2 bands were observed (IP: chicken IgG). After the lysates were immunoprecipitated with an antibody against HA, endogenous ROBO1 and ROBO2 were coimmunoprecipitated in the cells expressing HA-SLIT2 (IP: HA). All blots were cropped, and the gels were run under the same experimental conditions.

To test the direct physical interaction between SLIT2 and ROBO1 and/or ROBO2 in the GCs, coimmunoprecipitation and western blot analyses were conducted following the *SLIT2* overexpression assay. The immunoprecipitation of SLIT2 from the cells co-migrated with ROBO1 and ROBO2, while no co-migration with ROBO1 or ROBO2 was observed in the cell lysates immunoprecipitated with the control immunoglobulin G (IgG) antibody (Fig. 3). These data show that SLIT2 interacts with ROBO1 and ROBO2 simultaneously, which then activates its downstream mediator SRGAP1 in SLIT/ROBO signaling.

### SLIT2 down-regulates *FSHR*, *GDF9*, *STAR* and *CYP11A1* mRNA expression and suppresses granulosa cell proliferation

**Figure 4:**
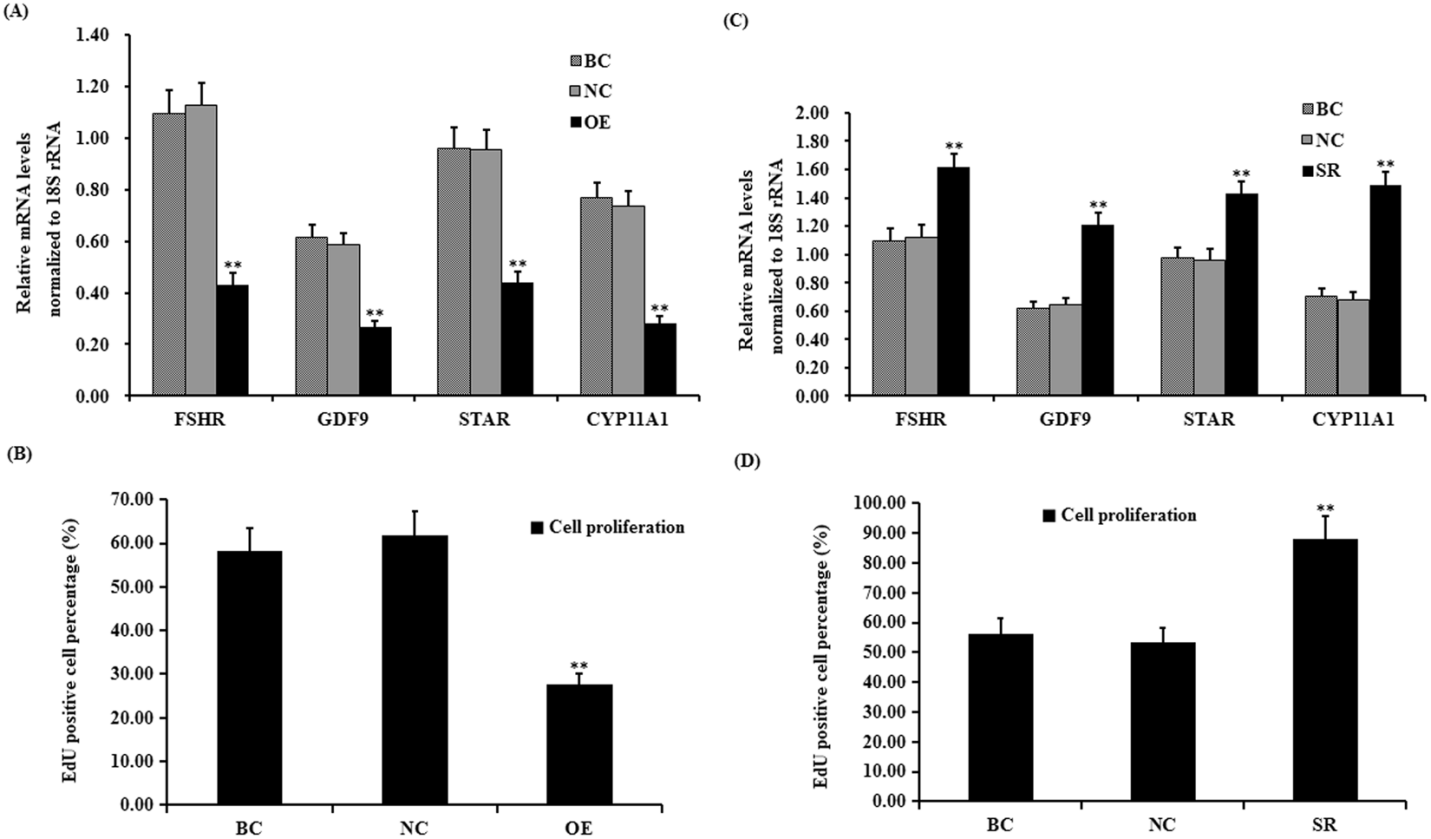
Effects of SLIT2 expression on biomarker gene expression and GC proliferation. (**A**) The granulosa cells were transfected with the reconstructed pYr-adshuttle-4-SLIT2 plasmids, a pYr-adshuttle-4 empty vector (negative control) or no plasmid (blank control). The expression of the biomarker genes *FSHR*, *GDF9, STAR* and *CYP11A1* before and after the GCs were transfected with the pYr-adshuttle-4-SLIT2 expression vector for 24 h was examined by qRT-PCR. The mRNA expression was normalized to that of the *18S rRNA* gene; the values on the bar graphs represent the mean ± SEM of 10 hens (n = 10) from a representative experiment. (**B**) GC proliferation was detected by an EdU incorporation assay. (**C**) The GCs were transfected with SLIT2 specific siRNAs, scrambled siRNA (negative control) or no siRNA (blank control). The expression of the biomarker genes in GCs transfected with or without the specific siRNA was examined by qRT-PCR. The mRNA expression was normalized to that of the 18S rRNA gene; the values on the bar graphs represent the mean ± SEM of 10 hens. (**D**) GC proliferation was detected by an EdU incorporation assay. For each group, the superscript symbol above the bar indicates that the difference was significant compared to the control group **P < 0.01, *P < 0.05.

As previously mentioned, a high expression of FSHR and GDF9 is required for GC proliferation and follicle selection^2,4–6^, and both STAR and CYP11A1 are key regulatory factors involved in the rate-limiting step of ovarian steroidogenesis and GC differentiation^6–8^. In this study, we investigated the influence of SLIT2 on *FSHR*, *GDF9*, *STAR* and *CYP11A1* mRNA expression in GCs and GC proliferation. As shown in Fig. 4, the mRNA expression levels of *FSHR, GDF9*, *STAR* and *CYP11A1* were significantly down-regulated in the cells with the SLIT2 overexpression (P < 0.01), and the cell proliferation ratios of the GCs were markedly decreased compared with those in the negative control (P < 0.01) (Fig. 4A,B, Supplemental Fig. S1A). These results indicate that SLIT2 plays an inhibitory role in GC proliferation and follicle selection that is consistent with the down-regulated *FSHR* and *GDF9* mRNA expression levels and may also suppress GC differentiation due to its negative regulation of *STAR* and *CYP11A1* mRNA expression during the prehierarchical follicle development stage.

To further confirm the suppressive effect of SLIT2 on GC proliferation, differentiation and follicle selection in cells transfected with the SLIT2-specific siRNA, the changes in *FSHR*, *GDF9*, *STAR* and *CYP11A1* mRNA expression and cell proliferation were investigated. As shown in Fig. 4C,D, the mRNA expression levels of *FSHR, GDF9*, *STAR* and *CYP11A1* were sharply enhanced in the cells after the transfection with the SLIT2-specific siRNA (P < 0.01). Due to its inhibitory role, the knock-down of SLIT2 expression attenuated its effect on GC proliferation, and the cell proliferation ratio in the GCs was remarkably increased compared with that in the negative control (P < 0.01) (see Supplementary Fig. S1B).

### ROBO1 and ROBO2 are necessary for the SLIT2-induced inhibition of granulosa cell proliferation

**Figure 5:**
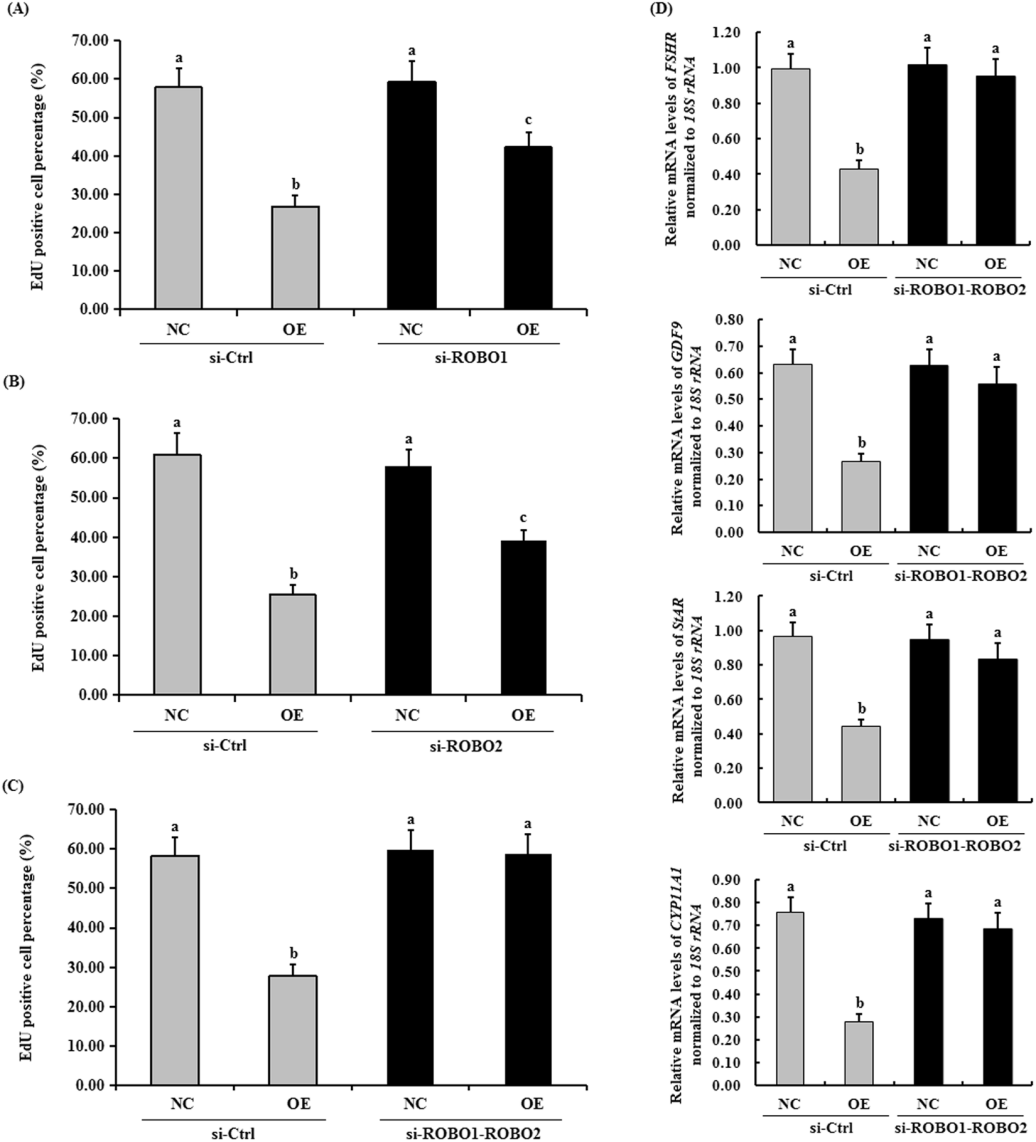
Effects of ROBO1/2 interference on biomarker gene expression and GC proliferation. In the GCs transfected with or without the reconstructed pYr-adshuttle-4-SLIT2 plasmids, the cells were co-transfected with specific siRNAs targeting the ROBO1 and/or ROBO2 genes. GC proliferation was examined by an EdU incorporation assay (original magnification × 20). (**A**) The effect of the ROBO1 interference on granulosa cell proliferation. (**B**) The effect of the ROBO2 interference on granulosa cell proliferation. (**C**) The effect of the ROBO1 and ROBO2 co-interference on granulosa cell proliferation. (**D**) The effect of the ROBO1 and ROBO2 co-interference on the mRNA expression levels of the biomarker genes *FSHR*, *GDF9, STAR* and *CYP11A1*. For each group, the different superscript above the bar indicates that the difference was significant (P < 0.01).

To determine which ROBO receptor is required for the SLIT2-induced inhibitory effect on granulosa cell proliferation, we knocked down endogenous ROBO1 and/or ROBO2 by using specific siRNA because both ROBO1 and ROBO2 interact with SLIT2. As shown in Fig. 5, in the GCs transfected exclusively with ROBO1 or ROBO2 siRNA for 24 h, the ROBO1 or ROBO2 knockdown partially abolished the suppressive effect of SLIT2 on GC proliferation (see Supplementary Fig. S2). Furthermore, the knockdown of both ROBO1 and ROBO2 completely abolished the inhibitory effects of SLIT2 on GC proliferation and the mRNA expression levels of *FSHR, GDF9*, *STAR* and *CYP11A1* (Fig. 5D).

### Regulation of the GTPase activity of CDC42 by the SLIT2 protein in GCs

**Figure 6:**
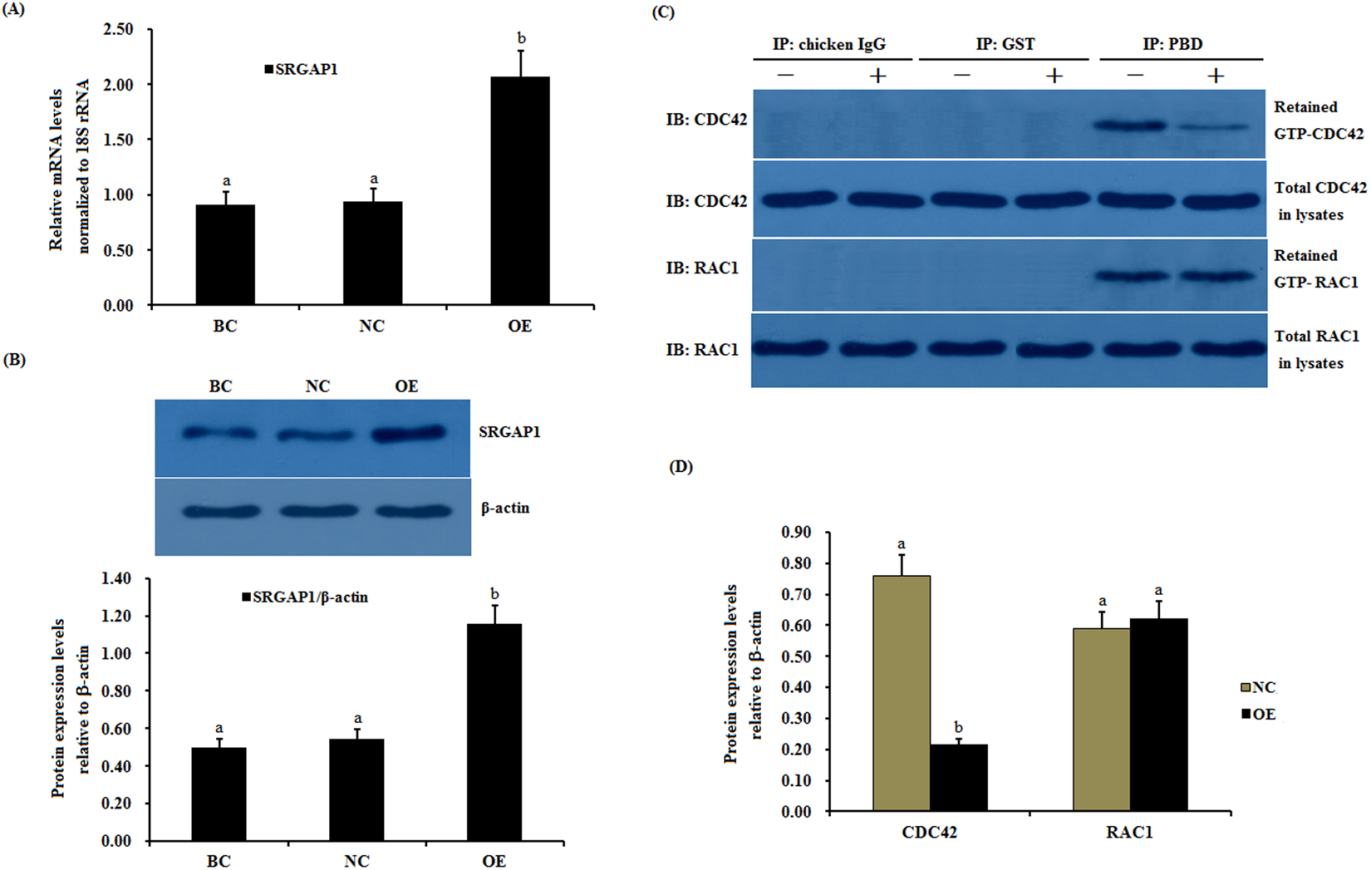
Effects of the overexpressed SLIT2 on *SRGAP1* expression and GTPase activity of CDC42 and RAC1 in GCs. The granulosa cells were transfected with the reconstructed pYr-adshuttle-4-SLIT2 plasmids, a pYr-adshuttle-4 empty vector (negative control) or no plasmid (blank control). (**A**) The expression of the *SRGAP1* gene before and after the GCs were transfected with the pYr-adshuttle-4-SLIT2 expression vector for 24 h was examined by qRT-PCR. The mRNA expression was normalized to that of the *18S rRNA* gene; the values on the bar graphs represent the mean ± SEM of 10 hens (n = 10) from a representative experiment. (**B**) The expression levels of the SRGAP1 protein in the GCs before and after the transfection with the pYr-adshuttle-4-SLIT2 vector was detected by western blotting. β-actin was used as a loading control. (**C**) The coimmunoprecipitation of CDC42 and RAC1 with the recombinant GST-PBD *in vitro*. The stimulation by the *SLIT2* overexpression of GTP-bound CDC42 and RAC1 in the granulosa cells from the prehierarchical follicles (6 to 8 mm in diameter) was examined by western blotting after performing a GST pull-down assay in which cell lysates were incubated with glutathione S-transferase (GST) and the recombinant GST-PBD. The immunoprecipitation (IP) with the PBD antibody was revealed by immunoblotting (IB) using monoclonal anti-CDC42 or anti-RAC1 antibodies (in the right-hand column). −, negative control; +, *SLIT2* overexpressed group. All blots were cropped, and the gels were run under the same experimental conditions. (**D**) The effects of the SLIT2 overexpression on the expression levels of GTP-bound CDC42 and RAC1 were detected by western blotting. β-actin was used as a loading control. For each group, the different superscript above the bar indicates that the difference was significant (P < 0.01).

**Figure 7:**
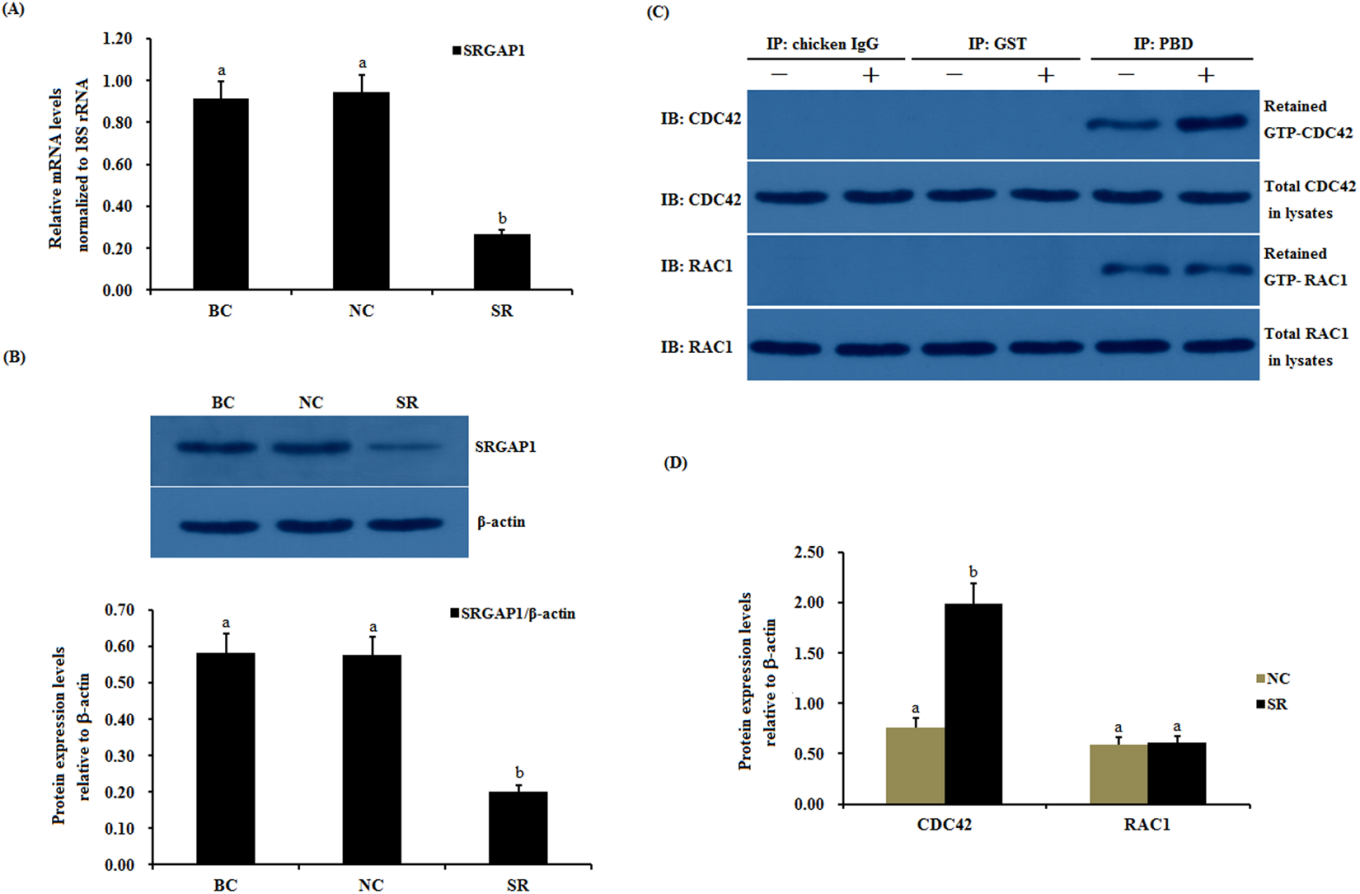
Effects of silencing *SLIT2* on *SRGAP1* expression and the GTPase activity of CDC42 and RAC1 in GCs. The granulosa cells were transfected with specific siRNAs targeting the *SLIT2* gene, scrambled siRNA (negative control) or no siRNA (blank control). (**A**) The expression of the *SRGAP1* gene before and after the GCs were transfected with the specific siRNA for 48 h was examined by qRT-PCR. The mRNA expression was normalized to that of the *18S rRNA* gene; the values on the bar graphs represent the mean ± SEM of 10 hens (n = 10) from a representative experiment. (**B**) The expression levels of the SRGAP1 protein in the GCs with and without specific siRNA interference (RNAi) was detected by western blotting. β-actin was used as a loading control. (**C**) The immunoprecipitation (IP) with the PBD antibody was revealed by immunoblotting (IB) using monoclonal anti-CDC42 or anti-RAC1 antibodies (in the right-hand column). −, negative control; +, *SLIT2* silencing group. All blots were cropped, and the gels were run under the same experimental conditions. (**D**) The expression levels of GTP-bound CDC42 and RAC1 under *SLIT2* silencing were determined by western blotting. β-actin was used as a loading control. For each group, the different superscript above the bar indicates that the difference was significant (P < 0.01).

Slit has been previously demonstrated to regulate the activities of endogenous Cdc42 by increasing the srGAP1-Robo1 interaction and inducing neuronal migration in the mouse brain^17^. Subsequently, we investigated the effects of SLIT2 on the activity of CDC42 and Rac1 Rho GTPases mediated by activated SRGAP1 in chicken ovarian GCs. As shown in Fig. 6, following the overexpression of SLIT2 *in vitro*, a remarkable increase in the mRNA and protein expression of *SRGAP1* was detected (p < 0.01) (Fig. 6A,B). In response to the elevated expression of SRGAP1, the expression level of the activated endogenous GTP-binding CDC42 protein was greatly down-regulated, and an elevation in the intrinsic GTPase activity on CDC42 was observed by stimulating GTP hydrolysis (p < 0.01), but no changes were detected in the RAC1 activity levels before and after the SLIT2 overexpression (Fig. 6C,D). Conversely, the mRNA and protein expression levels of *SRGAP1* were significantly down-regulated after SLIT2 was knocked down by siRNA in the ovarian GCs (p < 0.01). However, the activity of the CDC42 protein was markedly increased in the cells (p < 0.01, Fig. 7). These data demonstrate that the interaction between SLIT2 and its receptors ROBO1 and ROBO2 contributed to the repression of the activity of the GTPase CDC42 via SRGAP1, which may lead to the inhibition of the ser/thr kinase PAK and MAPK signaling pathway, which represents the downstream effector of the GTPase protein CDC42.

### Phosphorylation levels of MEK1/2 and ERK1/2 are affected by CDC42 and RAFs

**Figure 8:**
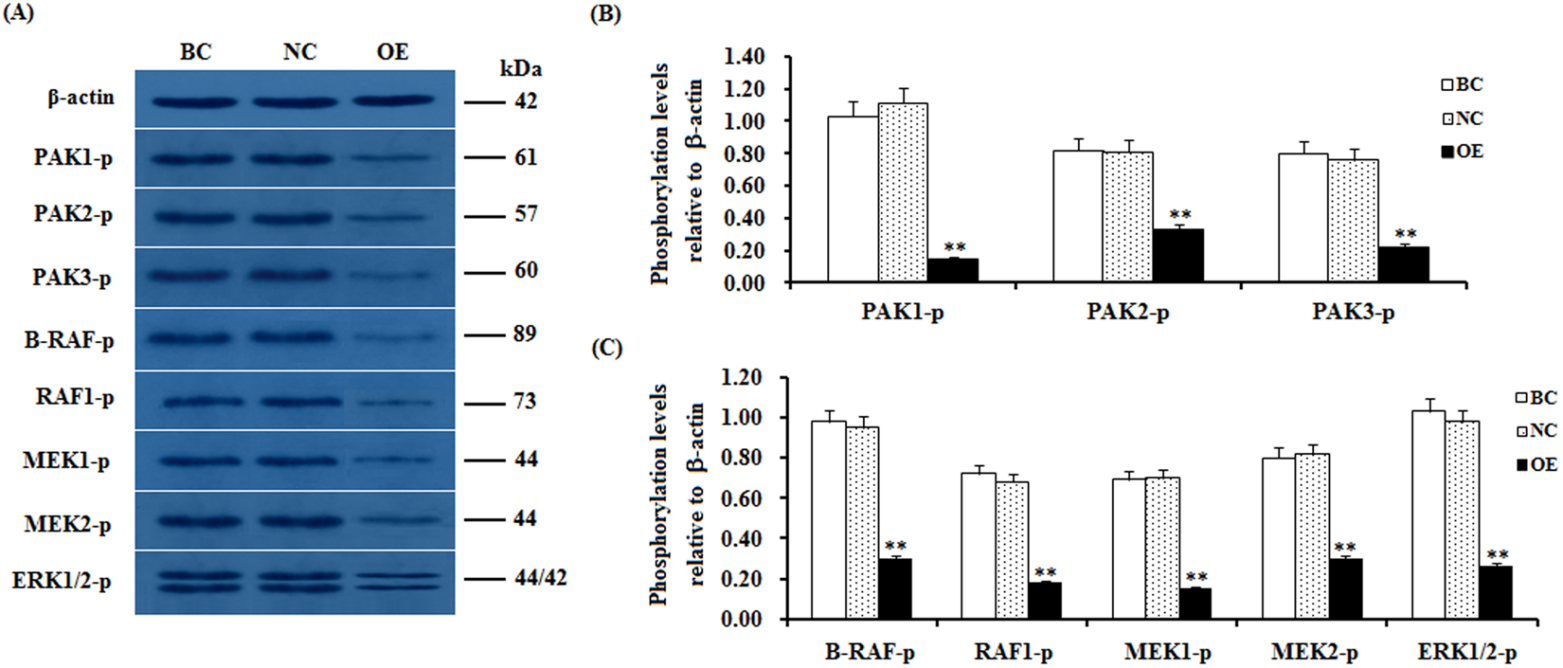
*SLIT2* overexpression-induced reduction in the phosphorylation levels of PAKs, RAFs and ERK1/2. The granulosa cells were transfected with the reconstructed pYr-adshuttle-4-SLIT2 plasmids, a pYr-adshuttle-4 empty vector (negative control) or no plasmid (blank control). (**A**) The immunoprecipitants were analyzed by western blotting for an *in vitro* phosphorylation assay. β-actin was used as a loading control. The blots were cropped, and the gels were run under the same experimental conditions. (**B**,**C**) The blotting signal intensity was quantified densitometrically after phosphorimaging (shown in **A**) and normalized for loading by comparison to the signal of β-actin. The signal intensity of the targeted proteins or phosphorylated proteins was expressed as a ratio to the β-actin signal in arbitrary units (n = 5 per mean ± SEM). Five independent experiments were carried out in triplicate. The results are representative of at least three independent experiments. significance is marked with different superscript symbols **P < 0.01, *P < 0.05.

**Figure 9:**
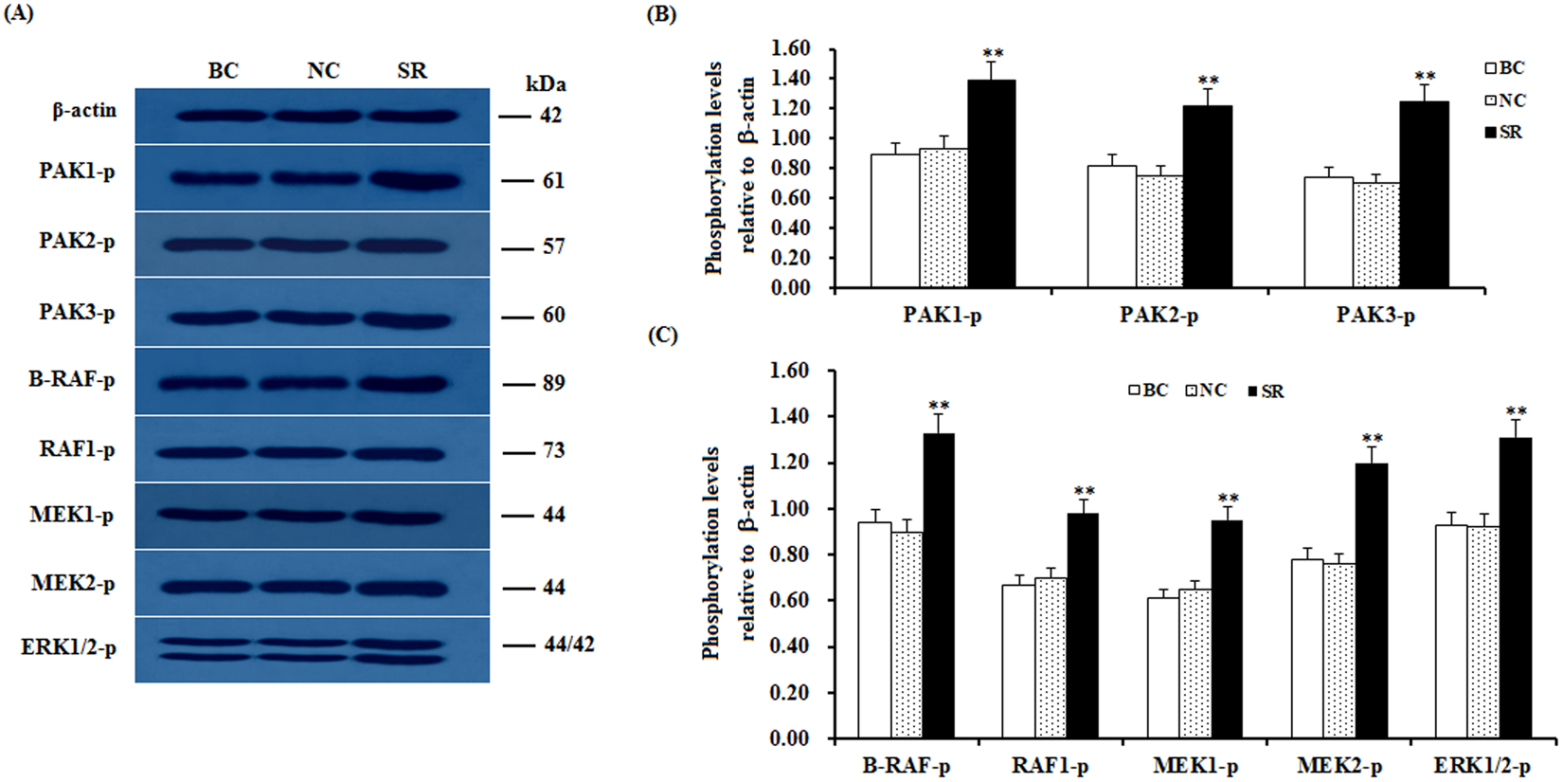
Knockdown of *SLIT2* enhances the phosphorylation levels of PAKs, RAFs and ERK1/2. The granulosa cells were transfected with the SLIT2-specific siRNA, scrambled siRNA (negative control) or no siRNA (blank control). (**A**) An *in vitro* phosphorylation assay was performed by western blotting. β-actin was used as a loading control. The blots were cropped, and the gels were run under the same experimental conditions. (**B**,**C**) The blotting signal intensity was quantified densitometrically after phosphorimaging (shown in **A**) and normalized for loading by comparison to the signal of β-actin. The signal intensity of the targeted proteins or phosphorylated proteins was expressed as a ratio to the β-actin signal in arbitrary units (n = 5 per mean ± SEM). Five independent experiments were carried out in triplicate. The results are representative of at least three independent experiments. The statistical significance is marked with different superscript symbols **P < 0.01, *P < 0.05.

**Figure 10:**
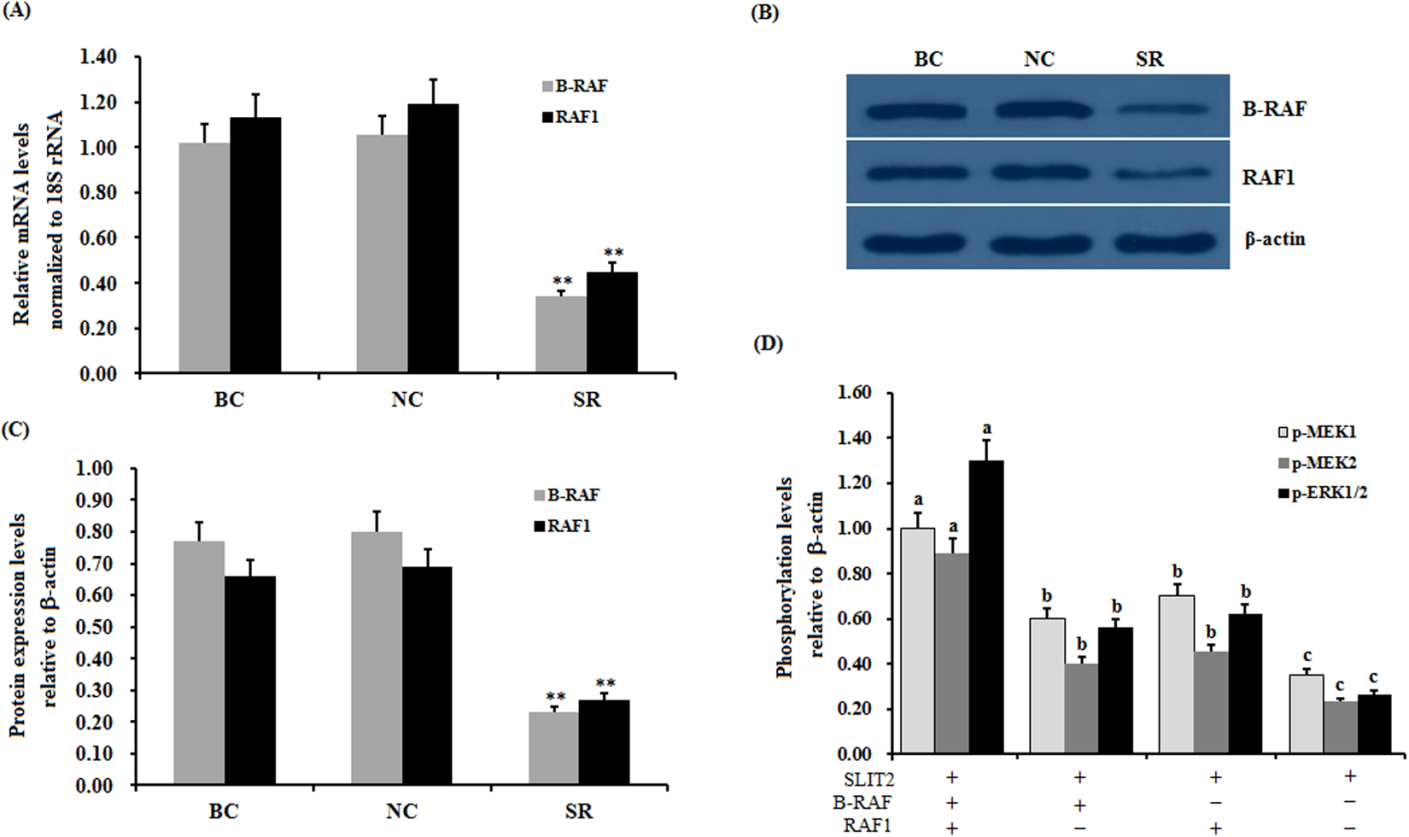
Effect of *RAF* RNAi on the SLIT2 overexpression-induced inhibition of the phosphorylation levels of MEK1/2 and ERK1/2. The granulosa cells were transfected with *B-RAF* and/or *RAF1* specific siRNA, scrambled siRNA (negative control) or no siRNA (blank control). (**A**) The expression of the *B-RAF* and *RAF1* genes before and after the GCs were transfected with specific siRNA for 48 h was examined by qRT-PCR. The mRNA expression was normalized to that of the *18S rRNA* gene; the values on the bar graphs represent the mean ± SEM of 10 hens (n = 10). (**B**) The immunoprecipitants were analyzed by western blotting. β-actin was used as a loading control. The blots were cropped, and the gels were run under the same experimental conditions. (**C**) The protein expression levels of B-RAF and RAF1 in the GCs with and without specific siRNA interference were detected by western blotting and normalized for loading by comparison to the signal of β-actin. (**D**) The granulosa cells were transfected with the reconstructed pYr-adshuttle-4-SLIT2 plasmids and co-infected with or without *B-RAF* and *RAF1* specific siRNA. The negative controls refer to the phosphorylation levels of MEK1/2 and ERK1/2 shown in Fig. 8. −, neither *SLIT2* overexpression nor knockdown of *RAFs*; +, *SLIT2* overexpression or knockdown of *RAFs*. The signal intensity of the phosphorylated proteins was expressed as a ratio to the β-actin signal in arbitrary units (n = 5 per mean ± SEM). The statistical significance is indicated with different superscript characters (P < 0.005).

To further determine the downstream target effectors of CDC42 and the regulatory mechanism underlying their effect on GC proliferation, differentiation and follicle selection, the activity level of the effectors in the PAKs and RAF-ERK1/2 MAPK signaling cascade was analyzed by western blotting. The experimental data show that after the CDC42 activity was significantly down-regulated by the SLIT2 overexpression in the GCs, the phosphorylation levels of the B-RAF, RAF1, MEK1/2 and ERK1/2 kinases exhibited a remarkable decrease accompanied by a large reduction in the phosphorylation levels of the PAK1, PAK2 and PAK3 proteins (p < 0.01, Fig. 8). Conversely, the RNAi-mediated knockdown of SLIT2 resulted in significantly enhanced phosphorylation levels of the B-RAF, RAF1, MEK1/2 and ERK1/2 kinases in the Cs (P < 0.01, Fig. 9). These results indicate that the inhibitory effect of SLIT2-ROBO1/2 on GC proliferation, differentiation and follicle selection is mediated by CDC42/PAK/RAFs and may lead to a decrease in MAPK/ERK activity. Furthermore, to probe the role of *B-RAF* and *RAF1* in the inhibition of the activities of the MEK1/2 and ERK1/2 kinases by SLIT2, B-RAF and RAF1 were knocked down in the GCs with *SLIT2* overexpression using RAF-specific RNAi (Fig. 10). The results show that when both *B-RAF* and *RAF1* are knocked down simultaneously, the inhibition of the activities of the MEK1/2 and ERK1/2 kinases by SLIT2 was largely blocked (p < 0.001); and these activities were partially blocked by the knockdown of *B-RAF* or *RAF1* alone (p < 0.001). Thus, B-RAF and RAF1 are essential for the SLIT2 suppression of the MAPK/ERK signaling cascade during follicle development.

### Regulatory roles of PAKs in SLIT2-induced GC proliferation and phosphorylation of B-RAF, RAF1, and ERK1/2

**Figure 11:**
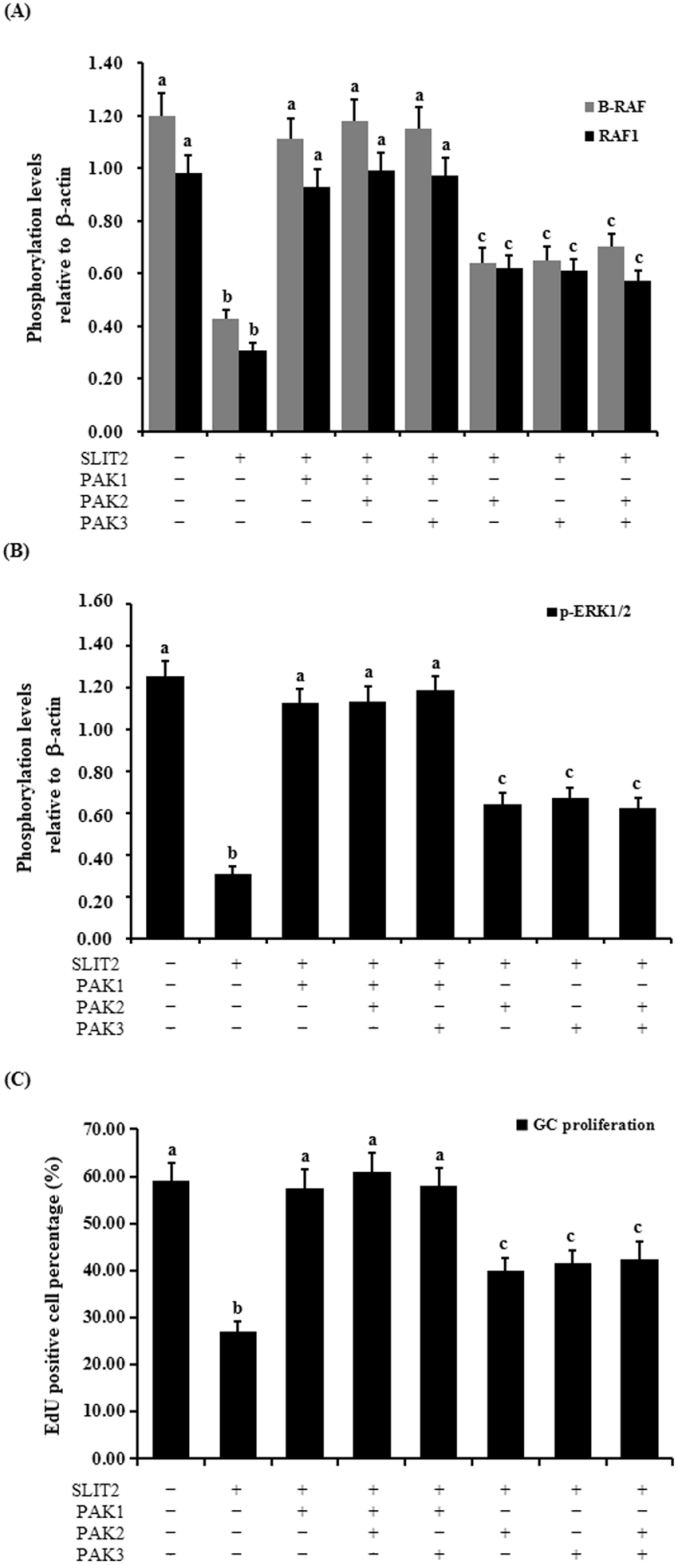
Regulation of the phosphorylation of B-RAF, RAF1, ERK1/2 and GC proliferation through the kinase PAKs. The granulosa cells were transfected with the reconstructed pYr-adshuttle-4-SLIT2 plasmids and co-infected with or without *PAK1*, *PAK2* and *PAK3* specific siRNA. (**A**) The phosphorylation levels of B-RAF and RAF1 proteins in the GCs with or without the specific siRNA interference were detected by western blotting and normalized for loading by comparison to the signal of β-actin. (**B**) The phosphorylation levels of the ERK1/2 proteins in the GCs with and without the specific siRNA interference were detected by western blotting and normalized for loading by comparison to the signal of β-actin. The signal intensity of the phosphorylated proteins was expressed as a ratio to the β-actin signal in arbitrary units (n = 10 per mean ± SEM). (**C**) Proliferation levels of the GCs with and without the specific siRNA interference were examined using an EdU Cell Proliferation Assay Kit. The statistical significance is indicated with different superscript characters (P < 0.01).

This experiment was conducted to further confirm the importance of the p21-activated kinases PAK1, PAK2 and PAK3 in the process of the SLIT2-induced inhibition of GC proliferation via the MAPK/ERK signaling pathway. As shown in Fig. 11, with the *SLIT2* overexpression, the phosphorylation levels of the B-RAF, RAF1 and ERK1/2 kinases were not remarkably changed by the transfection of the PAK1 siRNA in the GCs (p > 0.05). The phosphorylation levels of the B-RAF, RAF1 and ERK1/2 kinases were notably decreased by the transfection of the PAK2 and/or PAK3 siRNA in the GCs (p < 0.01), but the levels were higher than those of the PAK gene in the untransfected group (p < 0.05). Simultaneously, the repressive effect of SLIT2 on GC proliferation was significantly abrogated by the transfection of the PAK1 siRNA (p < 0.01), but this inhibition was partially prevented by the transfection of the PAK2 and/or PAK3 siRNA (p < 0.05). These results reveal that the inhibitory effect of SLIT2-ROBO1/2 on GC proliferation may be mainly mediated via CDC42/PAK1 by the inactivation of the RAF/MEK/ERK pathway during follicle growth and development in the chicken ovary.

## Discussion

The secreted SLIT2 glycoprotein is a component of the SLIT/ROBO signaling pathway that acts as a ligand of the transmembrane receptor(s) encoded by the ROBO gene(s); SLIT2 has been determined to be highly expressed in oocytes and granulosa cells of various-sized prehierarchical follicles during hen ovary development and follicles in ovine fetal and adult ovaries^10,11^. However, the SLIT/ROBO pathway has been identified to inhibit follicle formation, cell proliferation and cell migration and promote cell apoptosis in mammals^27^. Conversely, other published studies contradict these reports and document that the interaction between SLIT2 and the ROBO receptors may actually stimulate cell migration (such as in the human umbilical cord vascular endothelial cell) and induce angiogenesis in tumors^28^. Thus, the exact role and molecular regulatory mechanisms of SLIT2 that underpin the SLIT/ROBO signaling pathway in the prehierarchical follicular development of the hen ovary remain unclear. In the present study, the glycoprotein SLIT2 inhibited GC proliferation and decreased the mRNA and protein expression levels of *FSHR*, *GDF9*, *STAR* and *CYP11A1*, demonstrating that SLIT2 may suppress follicle selection, growth and maturation by hindering GC proliferation and differentiation during the prehierarchical follicular development of the hen ovary. Furthermore, the inhibitory effects of SLIT2 on GC proliferation and differentiation were mainly mediated by the direct physical interaction between the secreted SLIT2 and its receptor ROBO1 and/or ROBO2 of the four receptors (ROBO1–4) within the SLIT/ROBO pathway. Moreover, the expression levels of ROBO1 and ROBO2 are regulated by SLIT2 (Figs 1 and 2). The specific receptors ROBO1 and ROBO2 were confirmed to be required for the SLIT2-induced inhibition of GC proliferation and mRNA and protein expression of *FSHR*, *GDF9*, *STAR* and *CYP11A1*. These results suggest that the SLIT2-ROBO1 and/or ROBO2 signal act as a negative regulator of prehierarchical follicular growth and development in the hen ovary.

Chicken follicular GCs are well-known as key cells in the ovaries that undergo major morphological and physiological changes during the processes of folliculogenesis, maturation, ovulation and atresia. Many hormones, receptors (e.g., FSH and FSHR) and intraovarian factors, including GDF9, STAR and CYP11A1, are involved in these processes via various molecular mechanisms and signaling pathways. FSH has been shown to induce GC proliferation and differentiation by stimulating StAR expression and steroidogenesis^29^, and a relatively higher mRNA expression level of *FSHR* was observed in the GC layer of selected prehierarchical follicles^5^. FSHR knockdown induces porcine GC apoptosis and follicular atresia^30^. Although FSH and its receptor FSHR play a central role in the regulation of folliculogenesis, local ovarian factors can act to modulate (amplify or attenuate) FSH and FSHR action in an autocrine/paracrine manner during follicular growth and development. Among these factors, GDF9 can promote GC proliferation, which is critical for folliculogenesis after the primary stage, but simultaneously, GDF9 suppresses the FSH-induced differentiation of cultured GCs^4,31^. CYP11A1 is a key member of the cytochrome P450 superfamily of enzymes and is involved in GC proliferation, differentiation, and steroidogenesis by cleaving the side chain of cholesterol to produce pregnenolone, which is the first committed step and rate limiting process in steroid hormone synthesis^3,32^. All of these factors serve to regulate GC proliferation, differentiation and steroidogenesis, and understanding the regulatory mechanisms underlying the inhibitory effects of the SLIT2-ROBO1/ROBO2 signal on GC proliferation and the decrease in the mRNA and protein expression levels of *FSHR*, *GDF9*, *STAR* and *CYP11A1* was the focus in the current work and follow-up studies.

Researchers have shown that CDC42 and Rac1 are two members of the Rho GTPase family that perform a critical role in the regulation of a myriad of cellular functions, including mitosis, proliferation and apoptosis^16,19^. The Rho GTPases can interact with downstream effector molecules by functioning as molecular switches to propagate signal transduction in their GTP-loaded “on” state^33^. In mammals, Slit2 was found to interact with Robo1 to mediate repulsive cues in myogenesis^16^. The intracellular domain of Robo interacts with Rho GTPase activating proteins (GAPs), and two Slit-Robo GAPs (srGAPs) are expressed in regions responsive to Slit^17^. Subsequently, the activity of endogenous Cdc42 was down-regulated by increasing the srGAP1-Robo1 interaction, which induced neuronal migration in the mouse brain^17^. In the present study, a remarkable increase in *SRGAP1* mRNA and protein expression was detected following the induction of the overexpression of SLIT2 *in vitro* (p < 0.01). In response to the elevated expression of SRGAP1, the activity of the endogenous GTP-CDC42 protein was greatly decreased by the elevation in the intrinsic GTPase activity acting on CDC42 by stimulating GTP hydrolysis, but no changes were detected in the RAC1 activity levels before and after the SLIT2 overexpression. These results suggest that the inhibitory effect of SLIT2-ROBO1/2 on GC proliferation and differentiation may be mainly mediated by the inactivation of GTP-CDC42, which attenuates the activities of its downstream effectors by increasing the expression levels of the SRGAP1 protein in the chicken ovary.

The members of the MAPK family, including the B-RAF, RAF1, MEK1/2 and ERK1/2 kinases, have been previously shown to regulate diverse cellular functions, such as cell proliferation, differentiation, apoptosis and cell migration^34,35^. Furthermore, the Raf kinases play an important and specific role in the activation of the MAPK/ERK cascade^36^. For example, B-RAF can be activated by the cAMP/PKA pathway, which leads to increases in the levels of ERK-1/2 activity in supporting cells expressing B-Raf in the avian auditory epithelium^37^. The elevated expression levels of cAMP and B-Raf have been reported to increase the levels of cell proliferation in cultured neuronal cells^38^. However, the cAMP/PKA pathway inhibits Raf-1, thereby decreasing ERK-1/2 activity in cells that express only Raf-1^37^. cAMP has divergent effects on MAPK activity depending on whether the signaling occurs through Ras/Raf-1 or Rap1/B-raf^38^. To investigate whether the up-regulation of the SLIT2 glycoprotein is associated with the MAPK family, the phosphorylation levels of the B-RAF, RAF1, MEK1/2 and ERK1/2 kinases were determined by using western blotting. Our data demonstrate that the phosphorylation levels of the B-RAF, RAF1, MEK1/2 and ERK1/2 kinases were remarkably decreased, and a sharp decline in the phosphorylation levels of the proteins PAK1, PAK2 and PAK3 was observed (p < 0.01). These results suggest that the ERK MAPK family (B-RAF, RAF1, MEK1/2 and ERK1/2 kinases) might be involved in the SLIT2-SRGAP1-CDC42-induced inhibition of GC proliferation and differentiation, which is possibly mediated by the PAKs-B-RAF/RAF1 pathway. Further investigation showed that the down-regulation of the endogenous phosphorylation level of B-RAF and/or RAF1 by the transfection of the RAF siRNA into the GCs using Lipofectamine 2000 contributed to the significant decrease in the phosphorylated MEK1/2 and ERK1/2 protein levels. This result supports that B-RAF and RAF1 may play a critical role in the regulation of GC proliferation and differentiation that is mediated by the MEK1/2- ERK1/2 pathway in ovarian follicle selection.

**Figure 12:**
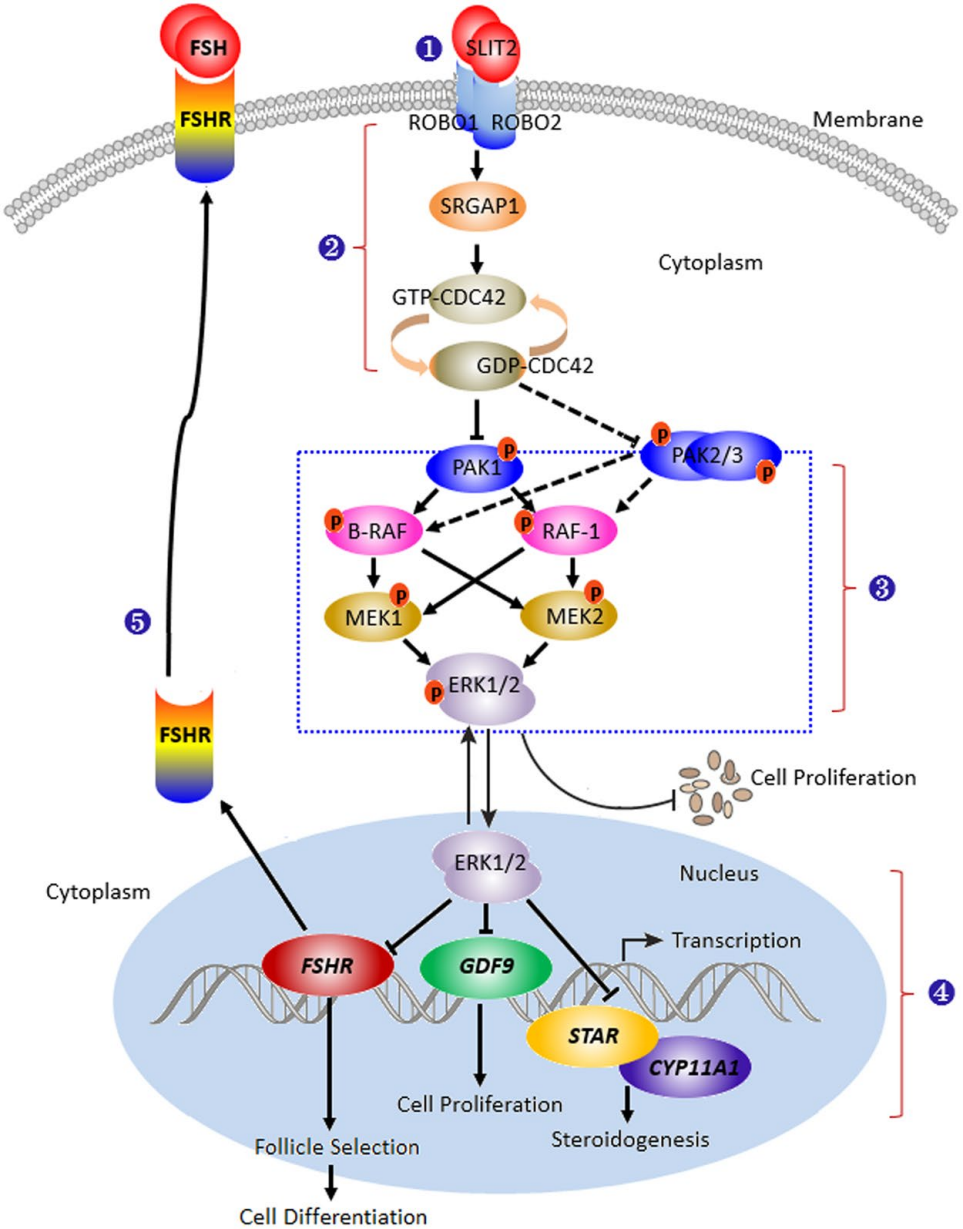
Schematic representation of the role of SLIT2-ROBO1/ROBO2 in the regulation of ovarian prehierarchical follicle growth and development mediated by the CDC42-RAFs-ERK1/2 MAPK cascade in hens. (**1**) The SLIT2 ligand interacts with ROBO1 and/or ROBO2 in an autocrine or paracrine manner and activates its downstream mediator SRGAP1 of SLIT/ROBO signaling. (**2**) Following the stimulation of the activated SRGAP1, the expression level of the activated endogenous GTP-binding CDC42 protein is down-regulated to enhance the intrinsic GTPase activity of CDC42 by stimulating GTP hydrolysis. Then, the activity levels of its downstream PAK effectors are attenuated, finally leading to the inhibitory effects of the MAPK/ERK signaling pathway on cell proliferation, differentiation and follicle selection. (**3**) The inhibitory effect of SLIT2-ROBO1/2 on GC proliferation, differentiation and follicle selection may be mainly mediated by CDC42/PAK1 via the MAPK/ERK1/2 signaling cascade and partially mediated by PAK2 and PAK3. (**4**) The effect of the inhibition of ERK1/2 induced by the interaction between SLIT2 and ROBO1/2 on the transcription of *FSHR*, *GDF9, STAR* and *CYP11A1* mRNA in the nucleus. (**5**) The reduced transcript and protein expression levels of *FSHR* may decrease the capacity of the FSHR to response to FSH. FSH-induced cell signaling via the protein kinase A/cyclic adenosine monophosphate (cAMP) pathway has been shown to be required for initiating differentiation in granulosa cells during follicle selection^5,7,9,49^.

In the regulation mediated by SRGAP1-CDC42, the PAKs function as major effectors of the small Rho GTPases Rac1 and Cdc42 downstream of the SLIT/ROBO pathway and play important roles in cell proliferation, apoptosis, survival and cell morphology^39^. CDC42 has been shown to be involved in the control of the epithelial cell cycle, proliferation and focal adhesion via the CDC42-PAK1-ERK1/2 signaling pathway^35^, and PAK1 and the serine/threonine-protein kinases PAK2 and PAK3 have been implicated in the regulation of cellular processes, such as gene transcription, cell apoptosis, morphology, and motility^40^. However, the exact roles and regulatory mechanisms of the chicken SLIT2-ROBO1/ROBO2 signal in the regulation of GC proliferation that is mediated by the SRGAP1-CDC42-PAKs pathway during ovarian follicular development remains unclear. The current data reveal that in the GCs with SLIT2 overexpression, the phosphorylation levels of the B-RAF, RAF1 and ERK1/2 kinases were not remarkably changed by the inhibition of PAK1 using siRNA interference in the GCs (p > 0.05); simultaneously, the repressive effect of SLIT2 on GC proliferation was significantly abrogated by the inhibition of PAK1 using siRNA (p < 0.01). Thus, the PAK1 kinase plays a pivotal role in transducing the SLIT2-SRGAP1-CDC42-induced inhibition of GC proliferation. Notably, compared with PAK1, the phosphorylation levels of the B-RAF, RAF1 and ERK1/2 kinases and GC proliferation levels were partially blocked by the PAK2 and/or PAK3 kinases. Thus, the inhibitory roles of SLIT2 in GC proliferation may be chiefly mediated by PAK1 via the RAF-MEK-ERK kinase regulatory cascade during ovarian prehierarchical follicle growth and development in hens. Although direct evidence proving that PAK2 and PAK3 are implicated in the regulation of the ovarian GC proliferation is lacking, PAK2 has been shown to bind and phosphorylate caspase-7, which fractionally decreases its activity and thereby inhibits cellular apoptosis in human breast carcinoma tissue^41^. Furthermore, PAK3 expression is required for cell migration and actin organization in transformed fibroblasts^42^. The over-expression of the PAK3 protein has been associated with the proliferation of mouse corticotrophs^43^. This study provided insight into the new roles of PAK2 and PAK3 involvement in promoting the activities of the RAFs/ERK MAPK pathway and GC proliferation by boosting the phosphorylation levels of the B-RAF, RAF1 and ERK1/2 kinases in chicken ovarian follicles; however, the inhibition of PAK2 and PAK3 using siRNA might simultaneously trigger their downstream effector proteins in GCs or be modulated by their specific upstream regulators via an unknown biofeedback bypass. Although the present data appear to suggest that PAK2 and PAK3 do not have a direct strong effect on GC proliferation via the ERK/MAPK pathway, evidence has shown that PAK1, PAK2 and PAK3 can play a compensatory role in proliferation after the loss of one of these proteins^44^. Collectively, based on the inhibitory effect of the SLIT2-ROBO1/ROBO2 signal on granulosa cell proliferation, differentiation and follicle selection, the core signaling component of the complex regulatory network implicated in the CDC42-PAK and ERK1/2 MAPK cascade in prehierarchical follicles in the chicken ovary is highlighted using a schematic representation (Fig. 12). These data suggest that the SLIT2-ROBO1/ROBO2 signal plays a negative regulatory role during prehierarchical follicular growth and development that is mediated by SRGAP1-CDC42-PAK and involves the ERK/MAPK pathway in the hen ovary.

In summary, this study is the first to reveal the suppressive role of SLIT2 in granulosa cell proliferation and the decreased mRNA and protein expression levels of *FSHR*, *GDF9*, *STAR* and *CYP11A1*. Moreover, the molecular mechanisms underlying the inhibitory effects induced by the SLIT2 ligand on granulosa cell proliferation, differentiation and follicle selection were revealed to involve the SLIT/ROBO pathway, which was transduced by CDC42-PAKs-ERK1/2 MAPK signaling for the homeostasis of the prehierarchical follicles during chicken ovary development.

## Materials and Methods

### Animals and sampling

The chickens sampled in this work were layers of the Lohmann brown commercial line that were reared in laying batteries according to our previously reported protocol^3^; all birds used in this experiment were obtained from the population and sacrificed at 21 weeks of age. Follicles of various sizes were collected from the hen ovaries according to the method reported by Stepińska and Olszańska^45^. A representative portion of each ovary was sampled, immediately frozen in liquid nitrogen and stored at −80 °C. Additionally, another equal part of the tissue was fixed using 4% neutral-buffered formalin at 4 °C. All procedures performed in the animals were approved by the Institutional Animal Care and Use Committee of Jilin Agricultural University (Changchun, China).

### Cell lines and cell culture

The culture of granulosa cells (GCs) from the hen prehierarchical follicles was performed according to previously published methods^7^. The cultured granulosa cells used in this experiment were purified and quantified. The identity of the granulosa cells was determined by H & E staining and a fluorescence staining analysis^3^.

### Quantitative real-time RT-PCR

To assess the mRNA expression of the target genes in the GCs, real-time quantitative reverse transcriptase PCR (qRT-PCR) was conducted according to our previously described method^3^. The primers used for the *SLIT2* gene were as follows: forward 5′-TGTAGGTGAACACTGCGATAT-3′ and reverse 5′-CCCTCTGGGCAAATACAAG-3′. The *18S rRNA* gene was used as an internal control in each reaction system as follows: forward 5′-TAGTTGGTGGAGCGATTTGTCT-3′ and reverse 5′-CGGACATCTAAGGGCATCACA-3′. The other primers utilized for the amplification of the genes, including *FSHR*, *GDF*9, *STAR* and *CYP11A1*, are listed in Table 1. Using the 2^−ΔΔCt^ method, the mRNA expression results were normalized against *18S rRNA* as an internal control.

**Table 1:** Primer pairs designed for the quantitative real-time PCR analysis.

Gene	Forward primer (5′ - 3′)	Reverse primer (5′ - 3′)	Accession No.	Size
*SLIT2*	TGTAGGTGAACACTGCGATAT	CCCTCTGGGCAAATACAAG	NM_001267075.1	111 bp
*ROBO1*	GACCCAAACTTGATTCCTAG	TGCCCTCACAAGGAATAGA	AF364047.1	243 bp
*ROBO2*	GGAGCCATTTACAGCAGC	TTGCCTTGGTCTGGGAGA	XM_416674.4	215 bp
*ROBO3*	GGAGGACGAGGAGATGAATG	AGTGTCCTCATGGGGCTTAG	XM_003642597.2	160 bp
*ROBO4*	GGGTGACATTGACTATGCTCTTGC	AGCCACAGCAGTGAGCCAGTT	XM_015298082.1	108 bp
*FSHR*	AATACCCTGCTAGGACTG	GAATACCCATTGGCTCA	NM_205079.1	238 bp
*GDF9*	ACTTTCACTCGGTGGATT	ATGCTGGGACATACTTGG	AY566700.2	175 bp
*STAR*	GCCAAAGACCATCATCAAC	TCCCTACTGTTAGCCCTGA	NM_204686.2	141 bp
*CYP11A1*	TCCGCTTTGCCTTGGAGTCTGTG	ATGAGGGTGACGGCGTCGATGAA	NM_001001756.1	112 bp
*SRGAP1*	ATTGCTGGTATTTACTCCT	TCTTGAACATTCTGGTGG	NM_001080101.1	132 bp
*B-RAF*	CAAACATCAACAACAGGGAC	CAGAAGCTCAATGGAGGC	NM_205302.1	182 bp
*RAF1*	TGTTTCCGCAAATACTGT	ATCCTGCTGGCACTGATC	NM_205307.2	257 bp
*18SrRNA*	TAGTTGGTGGAGCGATTTGTCT	CGGACATCTAAGGGCATCACA	AF173612.1	169 bp

### Construction of recombinant plasmids and cell transfection

The chicken *SLIT2* cDNA sequence (GenBank accession: NM_001267075.1) was amplified from a chicken cDNA library by PCR and cloned into a pUC57-Simple plasmid (Sangon Biotech Co., Ltd., Shanghai, China) by using the specific primers for the *SLIT2* gene as follows (see Supplementary Table S1): forward 5′-CCGCTCGAGCGGATGATGTGCGCCTGGGGGAGGCT-3′ and reverse 5′-AGCTTGTTTAAACGGCGCGCCGGTTAGGAGGGACAATTTGTACAGCC-3′; then, the sequence was subcloned into a pYr-adshuttle-4 expression vector containing an N-terminal hemagglutinin epitope (HA) tag (Wuhan Biobuffer Biotech Service Co. Ltd., China) to generate the pYr-adshuttle-4-SLIT2 expression construct.

The transfection for the *SLIT2* gene expression using the recombinant plasmid vector pYr-adshuttle-4-SLIT2 was performed as previously reported^4^. Briefly, the granulosa cells were randomly grouped and transfected with the pYr-adshuttle-4-SLIT2 plasmid and pYr-adshuttle-4 blank vector using the Lipofectamine 2000 transfection reagent (Invitrogen, Carlsbad, CA). The cultures (1 × 10^5^ cells/well in a 24-well plate) were conducted in a basal medium containing 1 μl/ml Polybrene (hexadimethrine bromide, Sigma) and incubated at 37 °C with 5% CO_2_. After 24 h of continuous culture, the granulosa cells were collected and lysed for the immunoblot and qRT-PCR analyses.

### Transfection of siRNA

Specific siRNAs targeting the *SLIT2* gene were designed using InvivoGen siRNA Wizard v3.1^46^. All siRNA sequences were blasted against the chicken genome database to eliminate cross-silencing phenomena with nontarget genes. The most effective *SLIT2-*specific siRNA was further screened by qRT-PCR and western blotting as follows: 5′-GGAAAUAACAUCACCAGAATT-3′. A scrambled siRNA that does not target any gene was used as the negative control siRNA as follows: 5′-UUCUCCGAACGUGUCACGUTT-3′. As mentioned above, the GCs were plated in 24-well plates, and the siRNAs were transfected into the culture cells using Lipofectamine 2000 (Invitrogen, Carlsbad, CA) according to the manufacturer’s instructions. The other siRNA sequences utilized for the interference of the targeted *ROBO1*/*2*, *PAKs* and *RAFs* genes are listed in Supplementary Table S2.

### Western blotting

Following the cell transfection, a western blot analysis of SLIT2, ROBO1/2, RAFs, CDC42 and RAC1 proteins and phosphorylated PAKs, RAFs, MEK1/2 and ERK1/2 kinases was conducted as previously described^3^ using total cellular extracts. Briefly, equivalent amounts of protein were separated by 10% (w/v) SDS-polyacrylamide gel under reducing conditions and electro-transferred to Protran nitrocellulose membranes (Whatman, Dassel, Germany). Affinity-purified antibodies against SLIT2 and the other proteins were used (Table S3). The samples were incubated with a horseradish peroxidase-conjugated anti-rabbit or anti-mouse IgG secondary antibody for 2 h at room temperature. The blots were subsequently incubated with the ECL western blotting reagent (Rockford, IL, USA) for 5 min and exposed to X-ray film for 1–5 min. The outcome was visualized by an ECL Plus western blotting detection system according to the manufacturer’s instructions. An anti-β-actin antibody (dilution 1:1000, Boster, Wuhan, China) was used as a loading control.

### Immunoprecipitation assay

As stated above, the GCs were transfected with the pYr-adshuttle-4-SLIT2 expression construct for 24 h in normal culture media and then lysed and immunoprecipitated in a buffer containing 50 mM Tris-HCl, pH 7.4, 150 mM NaCl, 0.2 mM PMSF, 1% Triton X-100 and 1 mM EDTA as previously described^47^. For the negative controls, the cell lysates were immunoprecipitated and incubated with chicken IgG (Sangon Co, Shanghai, China). The eluted samples or cell lysates were added to 4 × SDS sample buffer and heated at 95 °C for 5 min to denature the proteins. Then, the samples were subjected to SDS-PAGE for western blotting.

### GTPase activity assay

To test the SLIT2 regulation of the CDC42 and Rac1 activities mediated by the activated SRGAP1 in the GCs, the GTP-bound CDC42 and Rac1 were determined by specific binding to the p21-binding domain of PAK1 (PBD) according to previously described methods^22,23,48^ with a minor modification. In brief, GC lysates expressing the pYr-adshuttle-4-SLIT2 construct or interfering with the SLIT2-specific siRNA were incubated with 5 μg of the recombinant glutathione S-transferase (GST)-PBD (Sangon Co, Shanghai, China) and then conjugated with Glutathione Sepharose 4B beads (Beyotime Institute of Biotechnology) overnight. The beads were collected by centrifugation at 3,000 rpm for 3 min at 4 °C and washed three times with lysis buffer. Following boiling with 1 × SDS-PAGE protein loading buffer for 5 min, 20 μl of the supernatant were separated by SDS-PAGE and transferred to a PVDF membrane. After blocking with 5% skimmed milk at 4 °C, the membrane was incubated with the primary and secondary antibodies (HRP-goat anti-rabbit or anti-mouse IgG) and the PBD antibodies listed in Supplementary Table S3. The coimmunoprecipitated proteins were detected by western blotting. The whole-cell lysates were also analyzed for the presence of CDC42 for normalization.

### ***In vitro*** phosphorylation assay

Follicular GCs expressing the pYr-adshuttle-4-SLIT2 construct with or without the RAF-specific siRNA were lysed, and the cell lysates were immunoprecipitated using antibodies, including those against phosphorylated PAKs, B-RAF, RAF1, MEK1/2 and ERK1/2. The phosphorylation assay was conducted using previously reported procedures^4^. Briefly, the immunoprecipitants were resolved by 10% SDS-PAGE, and western blots were subsequently performed using the ECL western blotting reagent (Rockford, IL, USA) and primary and secondary antibodies (see Supplementary Table S3).

### EdU cell proliferation assay

Following the cell transfection for the SLIT2 overexpression or silencing, the variation in cell proliferation was determined by an EdU (5′-Ethynyl-2′-deoxyuridine) incorporation assay using a Cell-Light^TM^ EdU imaging kit (RiboBio, Guangzhou, China) according to the manufacturer’s protocol. Briefly, the control and transfected cells were seeded at a density of 1 × 10^5^ cells/well in a 96-well flat-bottom plate and cultured for 24 h. Then, the cells were incubated with 50 nM EdU for an additional 2 h at 37 °C. The cells were fixed with 4% formaldehyde for 15 min at room temperature and washed with glycine (2 mg/ml) for 5 min in a decolorization shaker; 0.5% Trion X-100 was added for 10 min, and the cells were washed with PBS three times. Then, the cells were incubated with a 1 × Apollo reaction cocktail (100 μl/well) for 30 min. The DNA was stained with 10 μg/ml of Hoechst 33342 (100 μl/well) for 20 min and visualized under a fluorescence microscope (Olympus, Tokyo, Japan). Each experiment was performed in triplicate and repeated five times. The number of EdU-positive cells was expressed as a percentage and calculated relative to the total number of cells counted in the microscope fluorescent image. The relative positive percentage was calculated as the average of five group values.

### Statistical analysis

The statistical calculations were performed using the SPSS 12.0 software package^4^. All experiments were repeated at least three times using different batches of sampled birds. To quantify the mRNA expression levels in the qRT-PCR analysis, four amplified products per bird from independent reactions were utilized. The data were collected and analyzed using a one-way ANOVA and Tukey’s multiple-comparison test if more than two groups were involved or Student’s *t-*test if the treatment and control groups were compared after confirmation of normal distributions for parametric analysis. *P* < 0.01 or *P* < 0.05 were considered statistically significant.

## Electronic supplementary material

Supplementary Information
