# Supplementary material for: Inhibitory effect of SLIT2 on granulosa cell proliferation mediated by the CDC42-PAKs-ERK1/2 MAPK pathway in the prehierarchical follicles of the chicken ovary
Source: Sci Rep. 2018 Jun 15;8:9168. doi: 10.1038/s41598-018-27601-z (PMC6003946; doi:10.1038/s41598-018-27601-z)

Inhibitory effect of SLIT2 on granulosa cell proliferation mediated by CDC42-PAKs-ERK1/2 MAPK pathway in the prehierarchical follicles of chicken ovary

Rifu Xu[1, 2, *, +], Ning Qin[1, 2, +], Xiaoxing Xu[1], Xue Sun[1], Xiaoxia Chen[1] & Jinghua Zhao[1]

1. Department of Animal Genetics, Breeding and Reproduction, College of Animal Science and Technology, Jilin Agricultural University, Changchun 130118, People's Republic of China

2. Key Laboratory of Animal Production and Product Quality Safety of Ministry of Education, Changchun 130118, People's Republic of China

***Corresponding author:**

Correspondence and requests for materials should be addressed to R. F. Xu (E-mail: poultryxu@jlau.edu.cn)

[+] **These authors contributed equally to this work.**

SUPPLEMENTARY INFORMATION FILE

Supplementary Table S1. Primer pairs used for the construction of the recombinant plasmid

Gene	Recombinant vector	Primer pairs (the forward and reverse, 5′ - 3′)	Restriction sites
SLIT2	pUC57-Simple-SLIT2	Forward: 5′-CCGCTCGAGCGG*ATGATGTGCGCCTGGGGGAGGCT*-3′	Xho I
		Reverse: 5′-AGCTTGTTTAAACGGCGCGCCGG*TTAGGAGGGACAATTTGTACAGCC*- 3′	Pme I

Note: The underlined nucleotides indicated the location corresponding to each of the restriction sites. The SLIT2 primers were shown in italics.

Supplementary Table S2. siRNA sequences selected for interference with the targeted genes

Target gene	siRNA sequence (the forward, 5′ - 3′)	Start position (nt)	Reference sequence
SLIT2	GGAAATAACATCACCAGAATT	546	NM_001267075.1
ROBO1	GTTGAACACCCATCTGATTTA	77	AF364047.1
ROBO2	GAGAACCAACAACCCTGAACT	910	XM_416674.4
B-RAF	GAGCATAACCCACCATCAATA	244	NM_205302.1
RAF1	GTGCGAAATGGGATGACCTTA	284	NM_205307.2
PAK1	GGAGTTGAAACAGAAGACAAA	111	NM_001162372.3
PAK2	GTTGCTACAGACCTCAAACAT	438	XM_004936995.2
PAK3	GAATCCTCAAGCAGTTCTAGA	526	XM_015278599.1

Supplementary Table S3. Antibodies used for the Western blot analysis

**Protein target**	**Primary antibody**	**Dilution used**	**Antibody type**	**Secondary antibody**	**Dilution used**	**Originated**
SLIT2	Mouse anti-SLIT2	1/1000	Monoclonal	anti-mouse IgG	1/1000	Sigma, St Louis, MO, USA
ROBO1	Mouse anti-ROBO1	1/2000	Monoclonal	anti-mouse IgG	1/1000	Sigma, St Louis, MO, USA
ROBO2	Mouse anti-ROBO2	1/2000	Monoclonal	anti-mouse IgG	1/1000	Sigma, St Louis, MO, USA
B-RAF	Rabbit anti-B-RAF	1/1000	polyclonal	anti-rabbit IgG	1/3000	Sangon Co, Shanghai, China
RAF1	Rabbit anti-RAF1	1/1000	polyclonal	anti-rabbit IgG	1/3000	Sangon Co, Shanghai, China
CDC42	Rabbit anti-CDC42	1/1000	Monoclonall	anti-rabbit IgG	1/2000	Rockford, IL, USA
RAC1	Rabbit anti-RAC1	1/1000	Monoclonal	anti-rabbit IgG	1/2000	Boster Biological Technology, China
PBD	Rabbit anti-pB-RAF	1/1000	polyclonal	anti-rabbit IgG	1/2000	Sangon Co, Shanghai, China
pPAK1	Mouse anti-pPAK1	1/2000	Monoclonal	anti-mouse IgG	1/3000	Sangon Co, Shanghai, China
pPAK2	Mouse anti-pPAK2	1/2000	Monoclonal	anti-mouse IgG	1/4000	Sangon Co, Shanghai, China
pPAK3	Mouse anti-pPAK3	1/2000	Monoclonal	anti-mouse IgG	1/3000	Sangon Co, Shanghai, China
pB-RAF	Rabbit anti-pB-RAF	1/1000	polyclonal	anti-rabbit IgG	1/3000	Sangon Co, Shanghai, China
pRAF1	Rabbit anti-pRAF1	1/1000	polyclonal	anti-rabbit IgG	1/3000	Sangon Co, Shanghai, China
pMEK1	Rabbit anti-pMEK1	1/2000	Monoclonal	anti-rabbit IgG	1/1000	Invitrogen, Carlsbad, CA, USA
pMEK2	Rabbit anti-pMEK2	1/2000	Monoclonal	anti-rabbit IgG	1/1000	Invitrogen, Carlsbad, CA, USA
pERK1/2	Rabbit anti-pERK1/2	1/2000	Monoclonal	anti-rabbit IgG	1/1000	Invitrogen, Carlsbad, CA, USA
β-actin	Mouse anti- β-actin	1/1000	polyclonal	anti-mouse IgG	1/2000	Boster Biological Technology, China

Supplementary Fig. S1. Effects of SLIT2 on GC proliferation in chicken ovaries

(A) The chicken granulosa cells were transfected with the reconstructed pYr-adshuttle-4-SLIT2 plasmids, a pYr-adshuttle-4 empty vector (negative control) or no plasmid (blank control). The effects of the SLIT2 overexpression on GC proliferation were detected by an EdU incorporation assay. All cell nuclei show blue fluorescence indicative of Hoechst 33342 staining; the EdU-labeled cells show red fluorescence indicating their newly synthesized DNA (original magnification ×20). (B) The GCs were transfected with SLIT2-specific siRNAs, scrambled siRNA (negative control) or no siRNA (blank control). The effects of the SLIT2 silencing on GC proliferation were detected by an EdU incorporation assay (original magnification ×20). BC, blank control; NC, negative control; OE, SLIT2 overexpression group; SR, SLIT2 knockdown group.

(A)

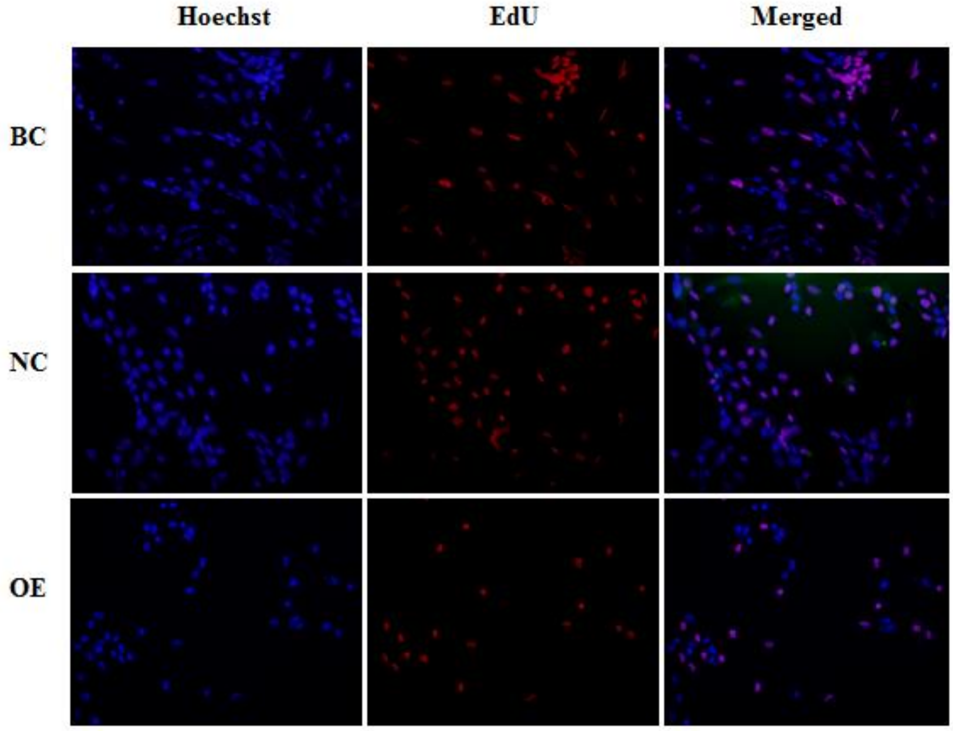

(B)

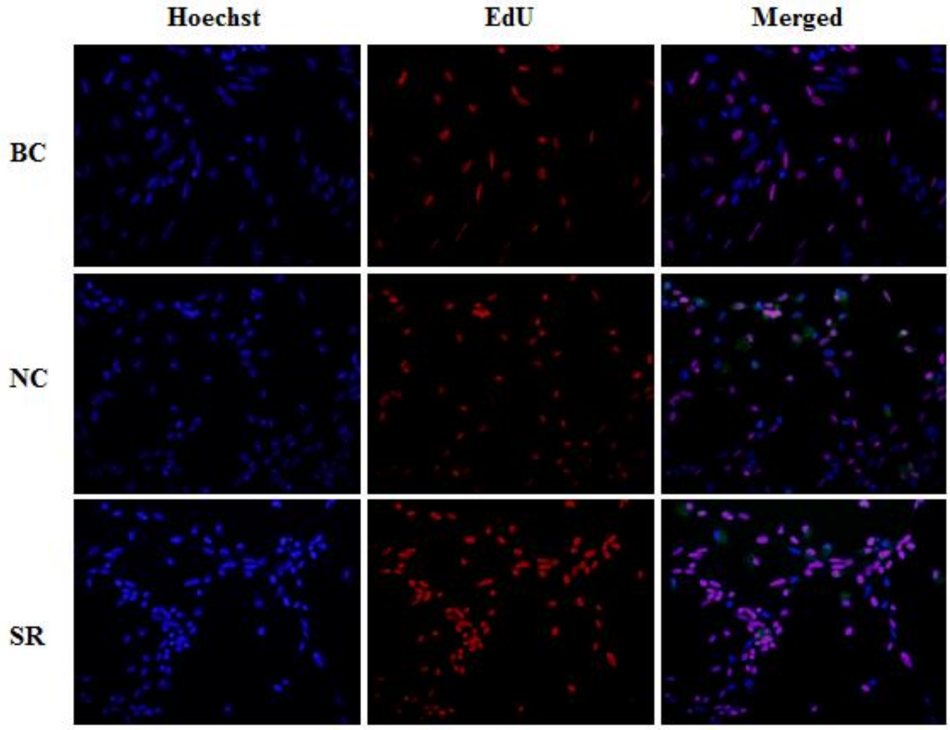

Supplementary Fig. S2. Effects of ROBO1 and/or ROBO2 on the SLIT2-induced inhibition of granulosa cell proliferation

In the GCs transfected with or without the reconstructed pYr-adshuttle-4-SLIT2 plasmids, the cells were co-transfected with specific ROBO1 and/or ROBO2 siRNAs. (A) GCs transfected exclusively with or without the reconstructed pYr-adshuttle-4-SLIT2 plasmids, (B) co-transfected with specific ROBO1 siRNA, (C) co-transfected with specific ROBO2 siRNA, or (D) co-transfected with both the ROBO1 and ROBO2 siRNAs. GC proliferation was examined by an EdU incorporation assay (original magnification ×20). NC, negative control; OE, SLIT2 overexpression group.

(A)

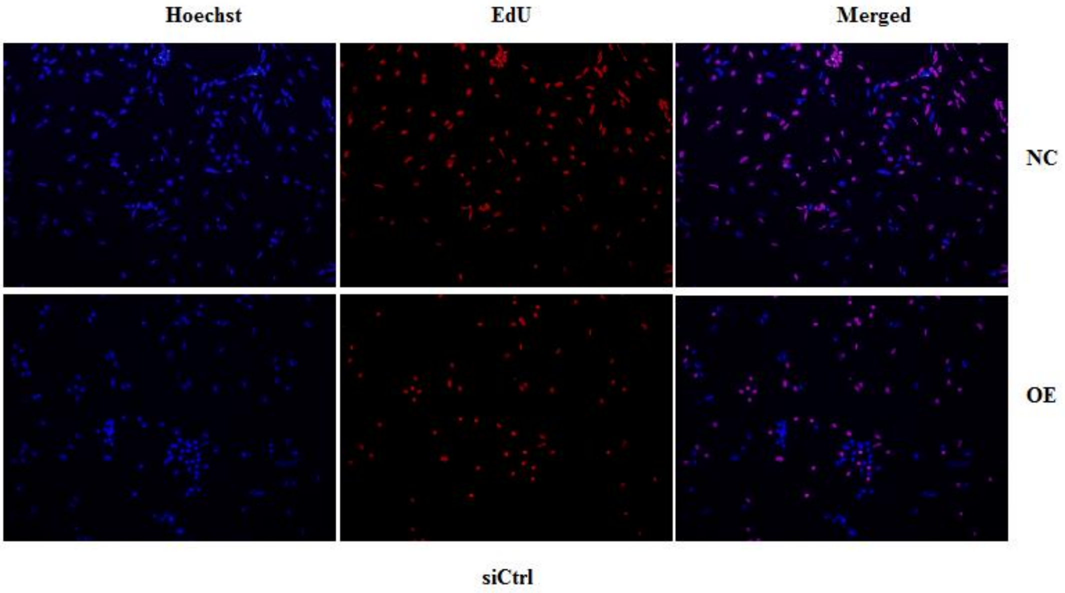

(B)

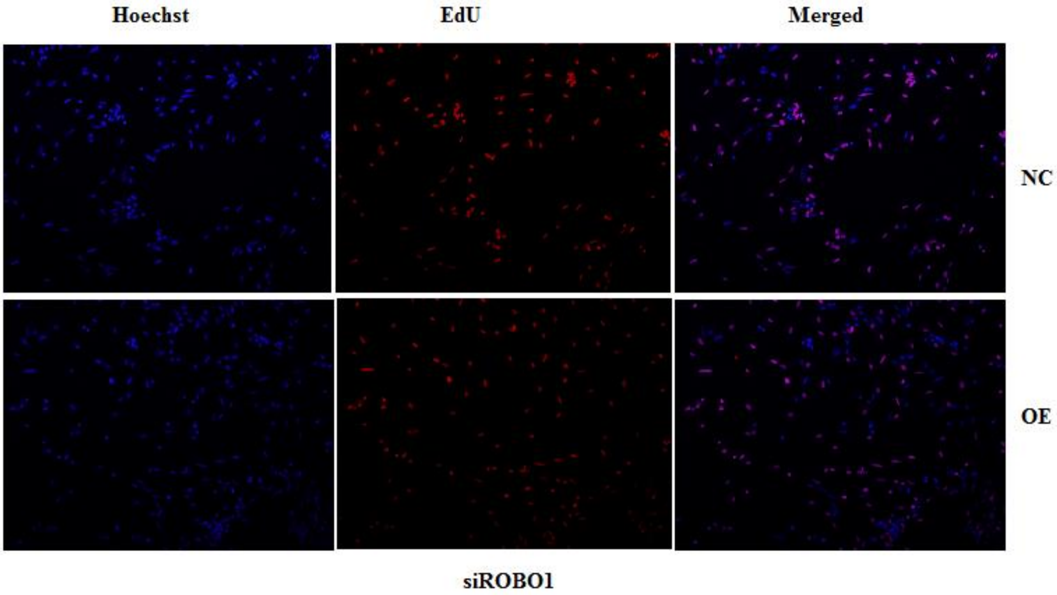

(C)

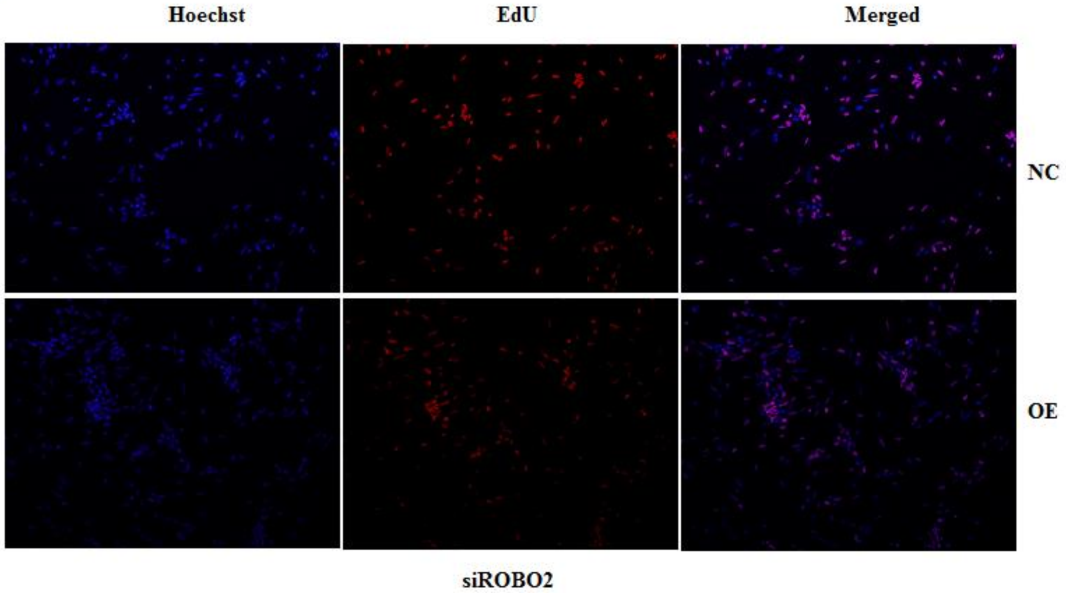
Hoechst
EdU
Merged
NC
OE
siROBO2

(D)

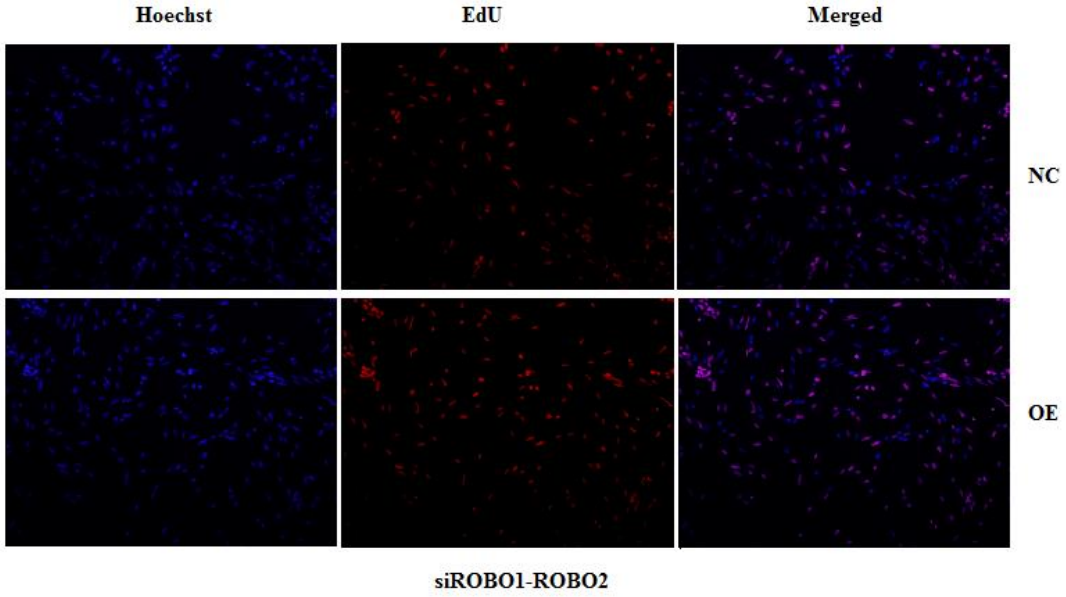
Hoechst
EdU
Merged
NC
OE
siROBO1-ROBO2

Supplementary Figure S3.
Original blot images in Figures 1, 2, 3, 6, 7, 8, 9 and 10.

Original images for SLIT2, ROBO1, ROBO2 and β-actin expression in the GCs before and after the transfection with the pYr-adshuttle-4-SLIT2 vector detected by western blotting. The manipulated versions are reported in Fig. 1 of the manuscript.

Fig.1B-1 full blot image of SLIT2

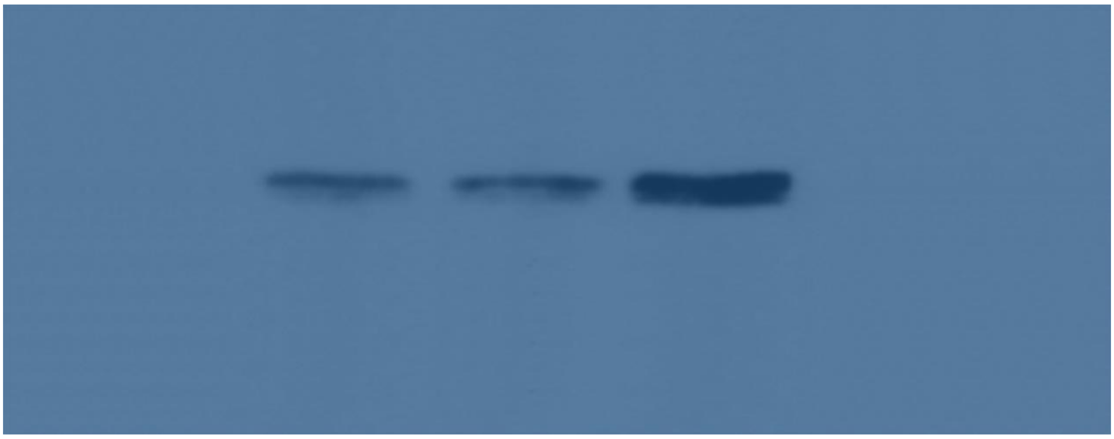

Fig.1B-2 full blot image of beta-actin

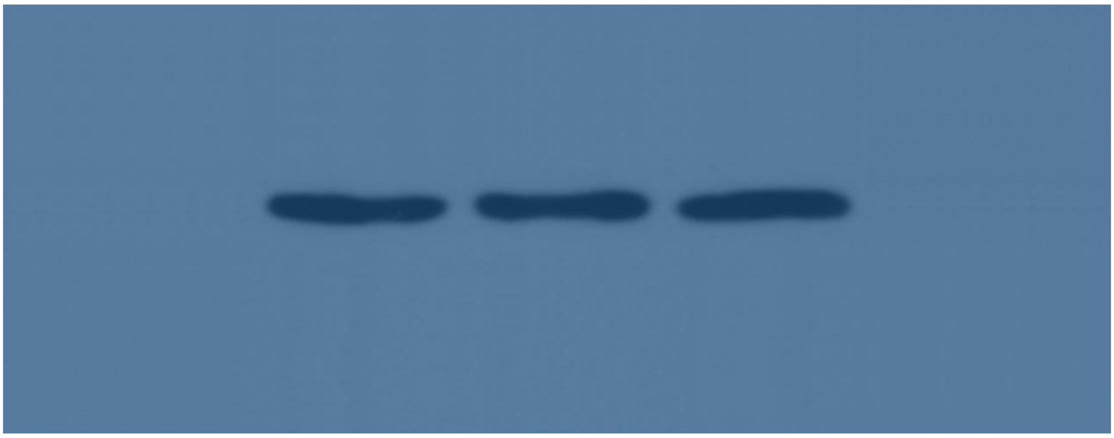

Fig.1D-1 full blot image of ROBO1

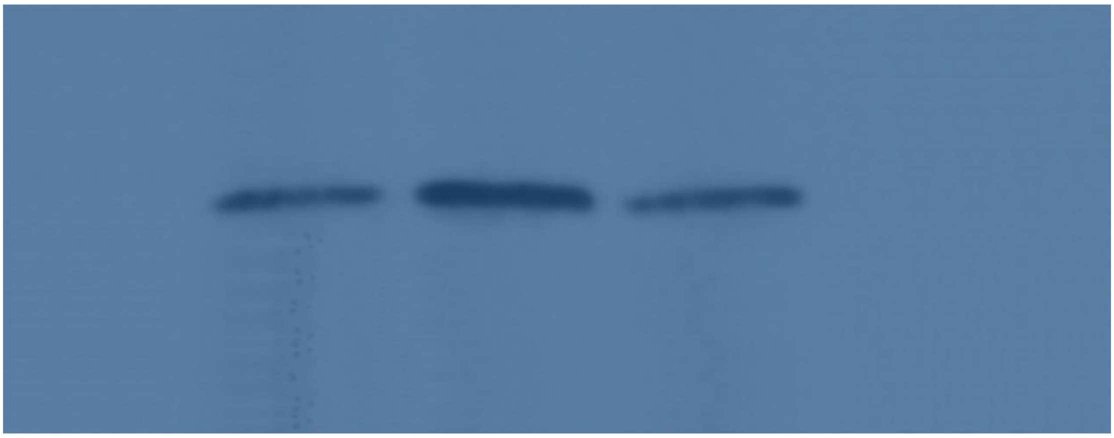

Fig.1D-2 full blot image of ROBO2

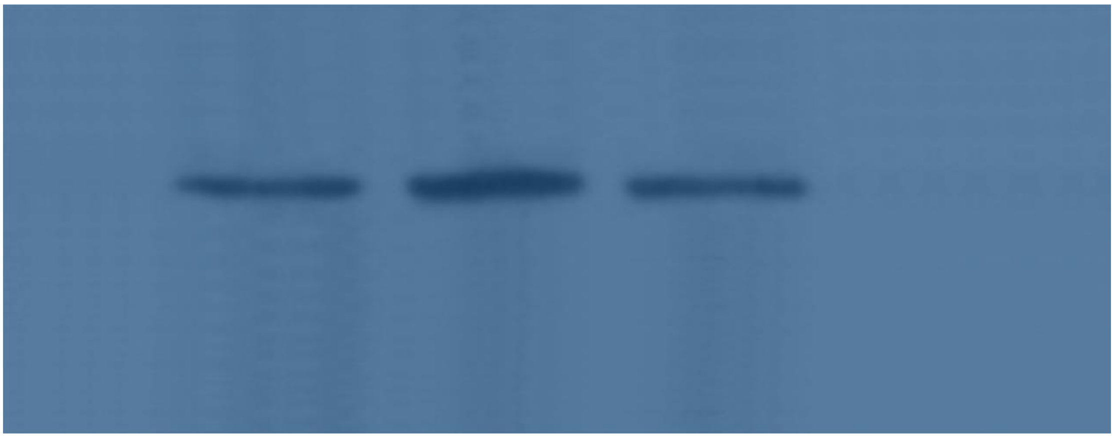

Fig.1D-3 full blot image of beta-actin

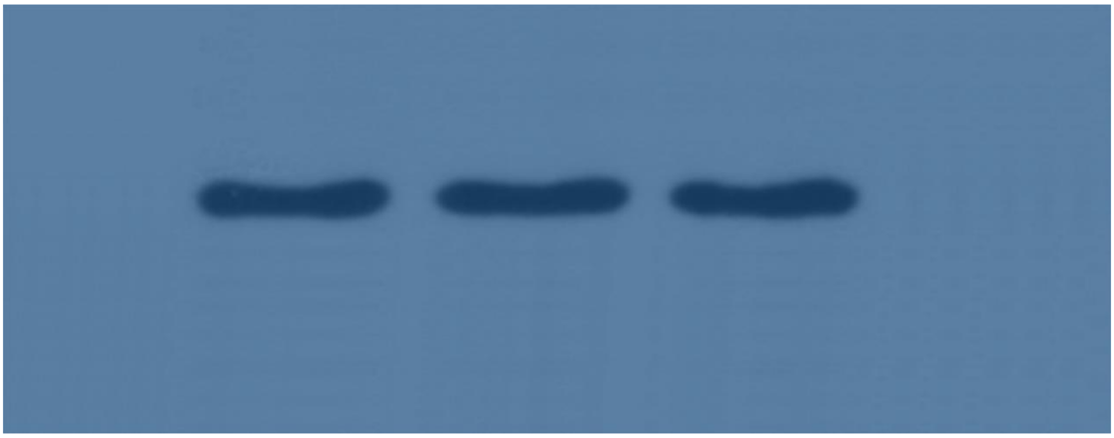

Original images for SLIT2, ROBO1, ROBO2 and β-actin expression in the GCs before and after the transfection with the SLIT2-specific siRNA. The manipulated versions are reported in Fig. 2 of the manuscript.

Fig.2B-1 full blot image of SLIT2

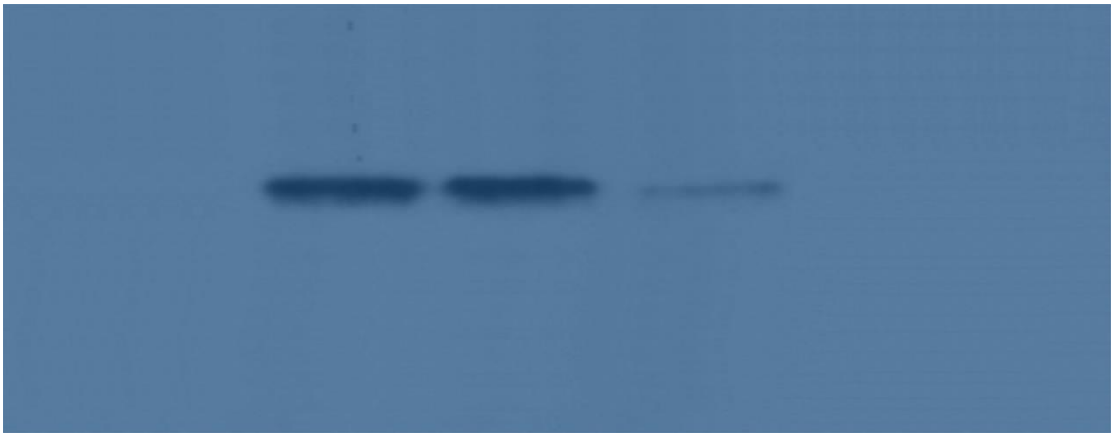

Fig.2B-2 full blot image of beta-actin

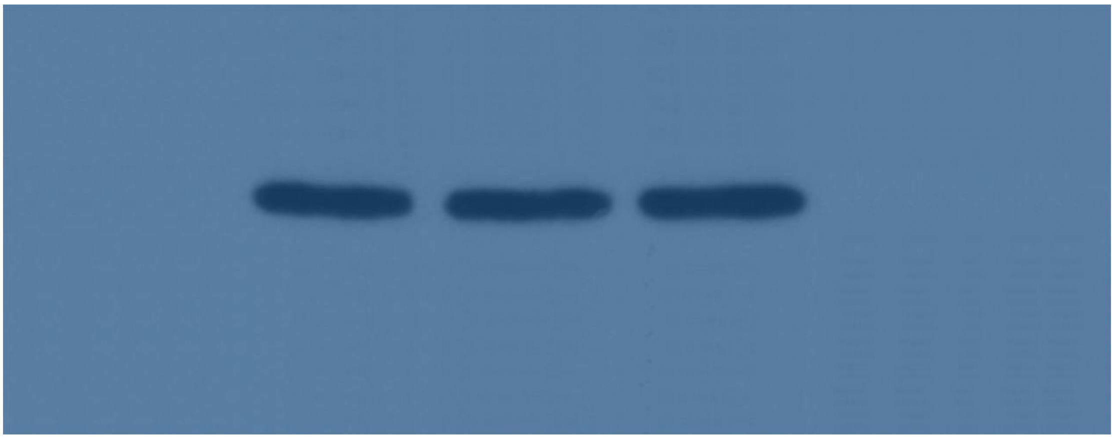

Fig.2D-1 full blot image of ROBO1

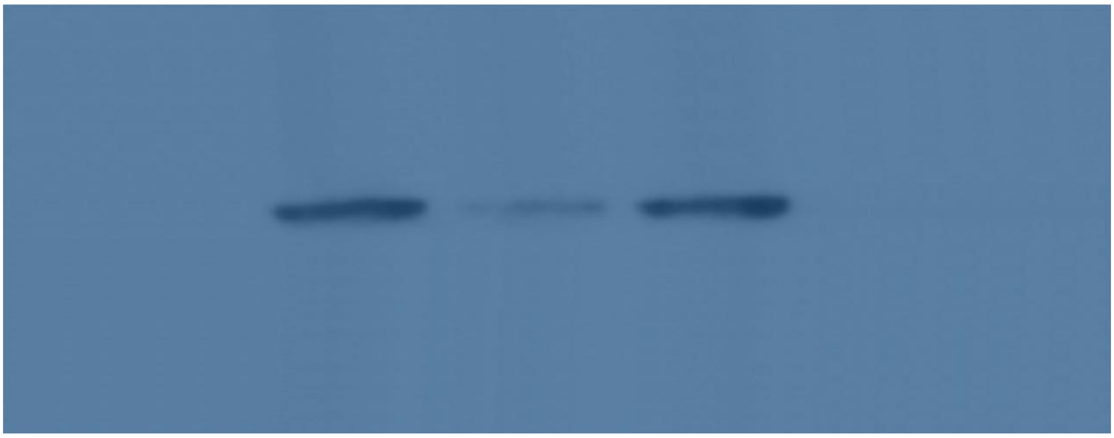

Fig.2D-2 full blot image of ROBO2

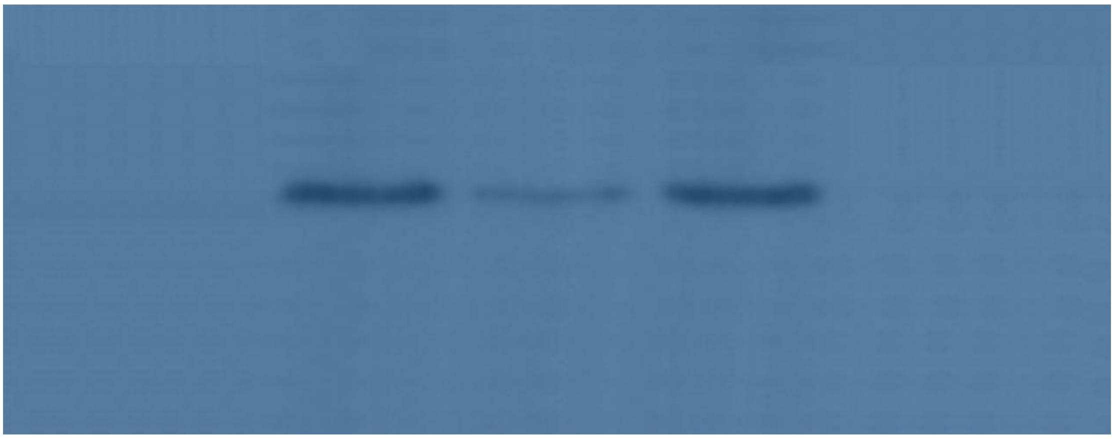

Fig.2D-3 full blot image of beta-actin

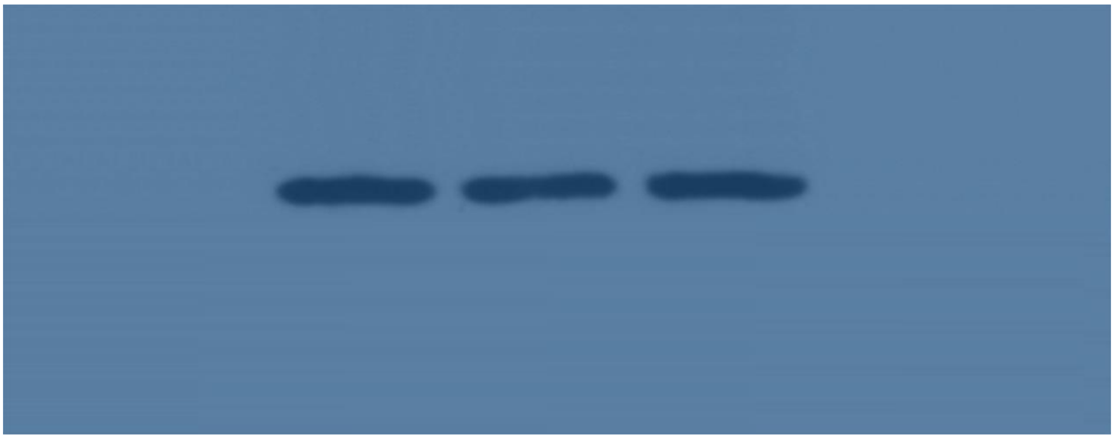

Original images for SLIT2, ROBO1 and ROBO2 protein in the GCs before and after the transfection with the pYr-adshuttle-4-SLIT2 construct determined by the coimmunoprecipitation experiment. The manipulated versions are reported in Fig. 3 of the manuscript.

Fig.3-1 full blot image of SLIT2

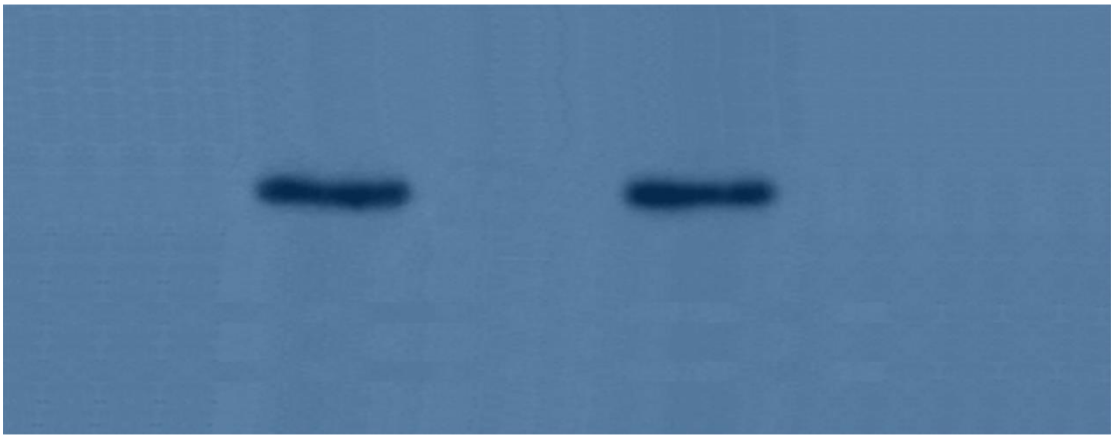

Fig.3-2 full blot image of ROBO1

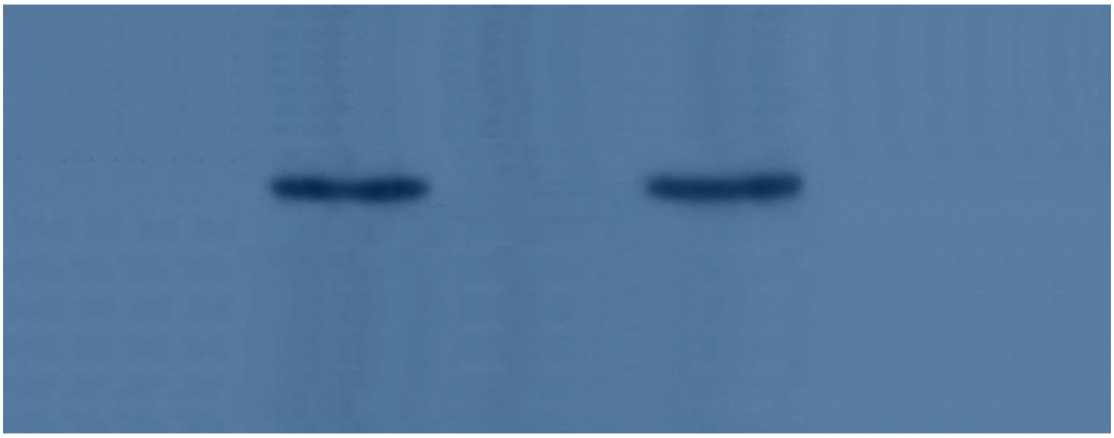

Fig.3-3 full blot image of ROBO2

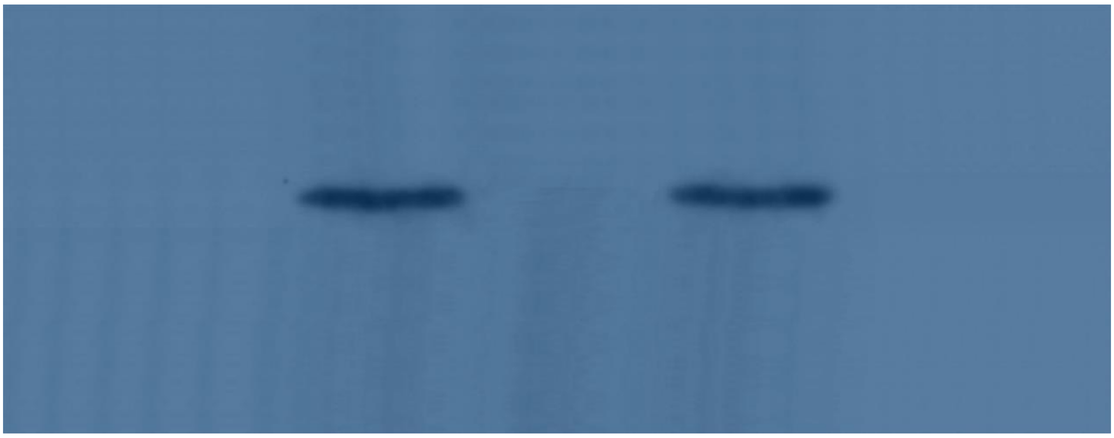

Original images for SRGAP1, GTP-CDC42, CDC42, GTP-RAC1, RAC1 and β-actin expression in the GCs before and after the transfection with the pYr-adshuttle-4-SLIT2 expression construct determined by the immunoblotting. The manipulated versions are reported in Fig. 6 of the manuscript.

Fig.6B-1 full blot image of SRGAP1

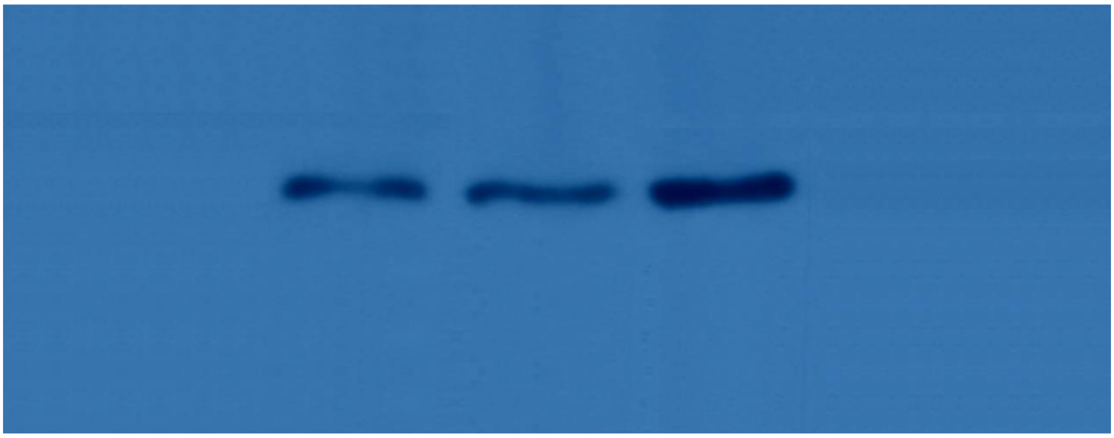

Fig.6B-2 full blot image of beta-actin

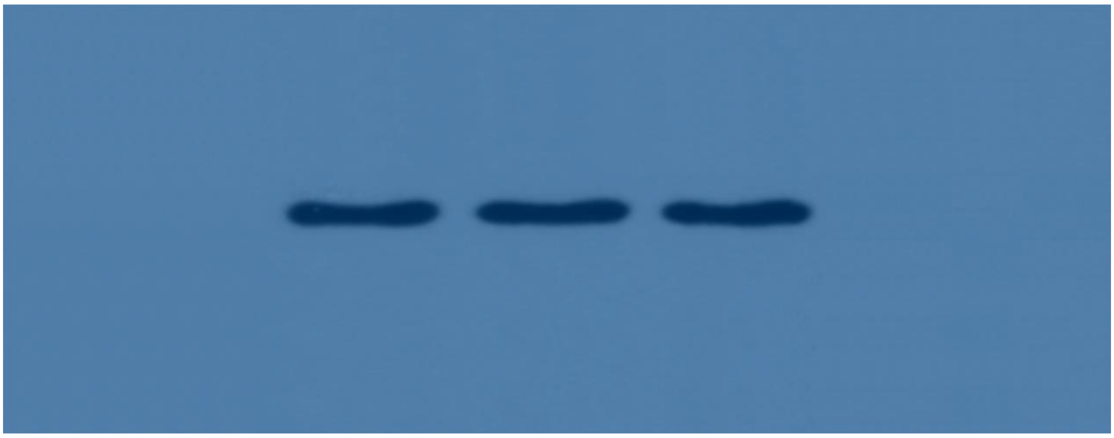

Fig.6C-1 full blot image of GTP-CDC42

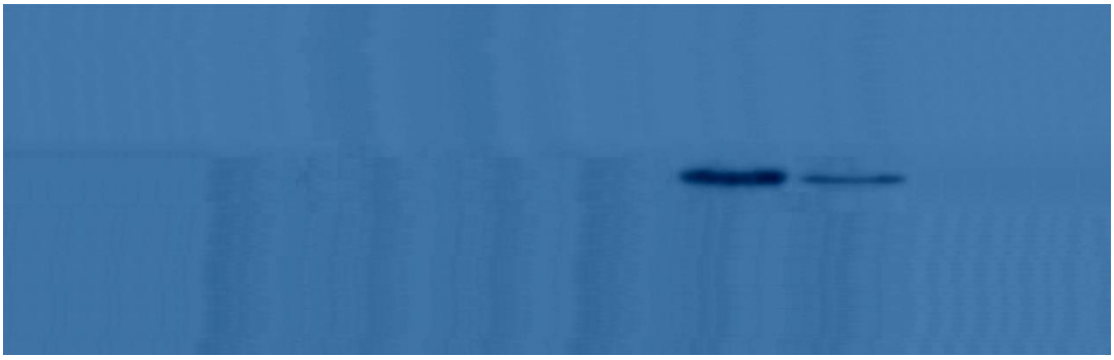

Fig.6C-2 full blot image of total CDC42

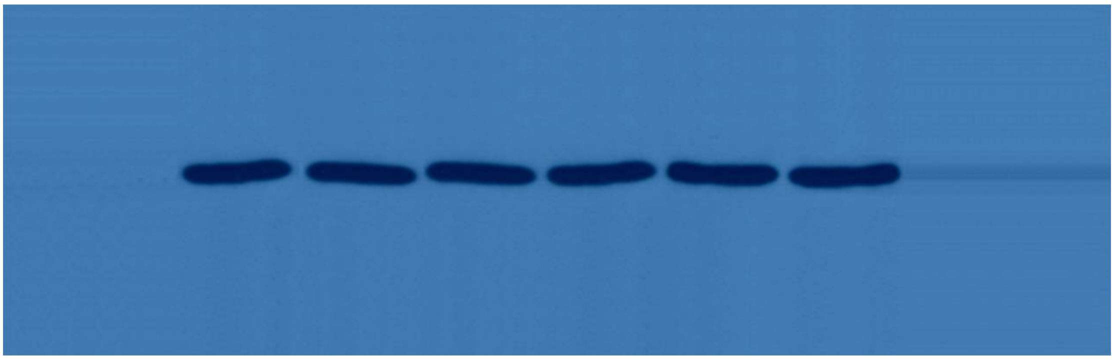

Fig.6C-3 full blot image of GTP-RAC1

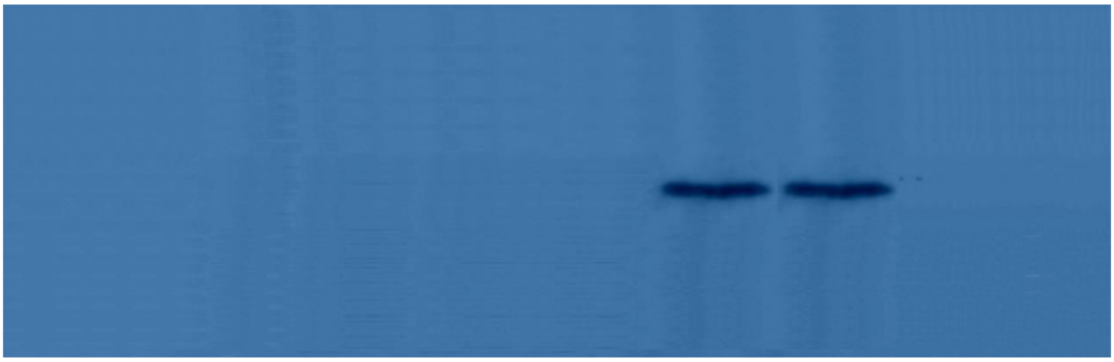

Fig.6C-4 full blot image of total RAC1

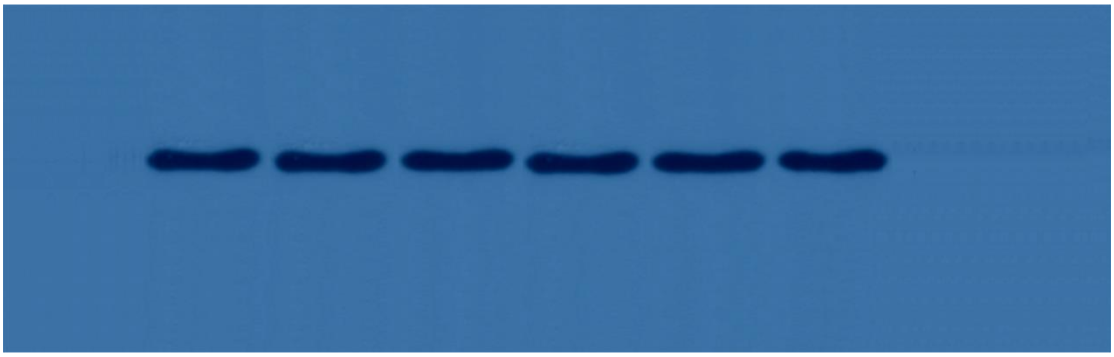

Original images for SRGAP1, GTP-CDC42, CDC42, GTP-RAC1, RAC1 and β-actin expression in the GCs before and after the transfection with the specific siRNAs targeting SLIT2 gene tested by the immunoblotting. The manipulated versions are reported in Fig. 7 of the manuscript.

Fig.7B-1 full blot image of SRGAP1

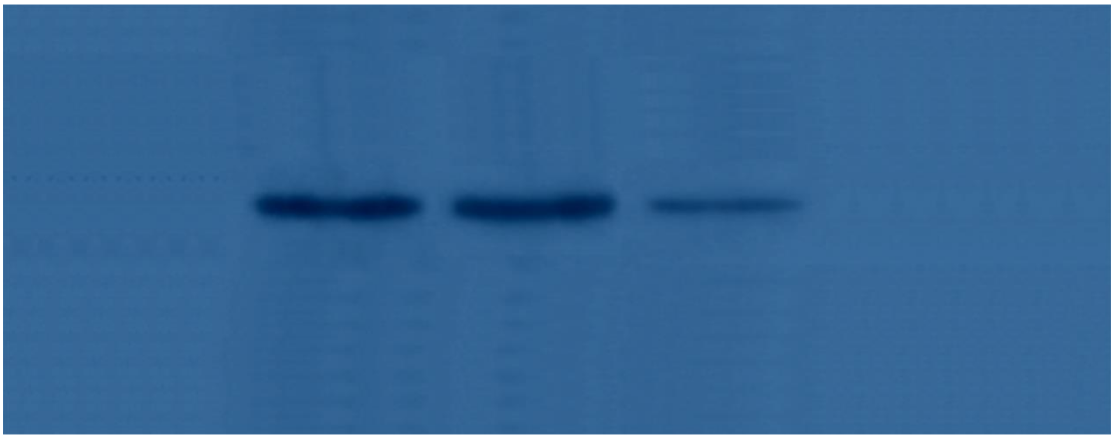

Fig.7B-2 full blot image of beta-actin

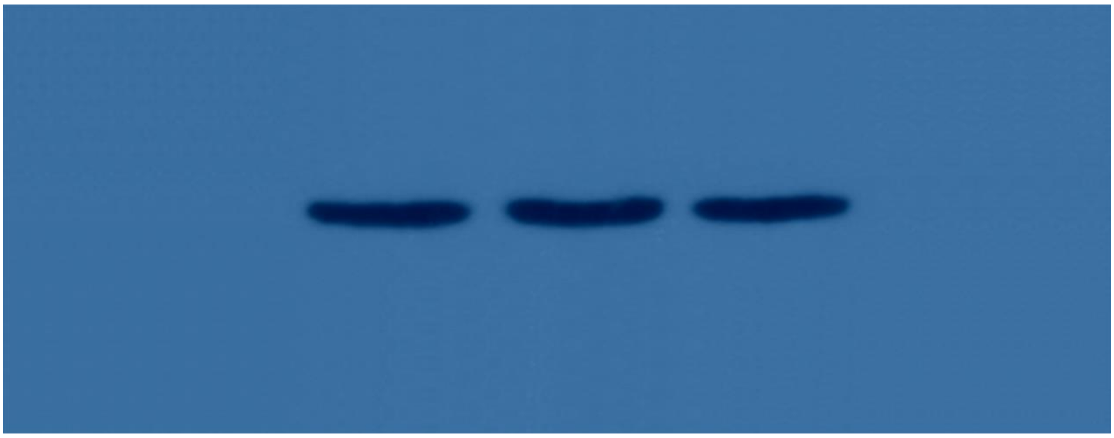

Fig.7C-1 full blot image of GTP-CDC42

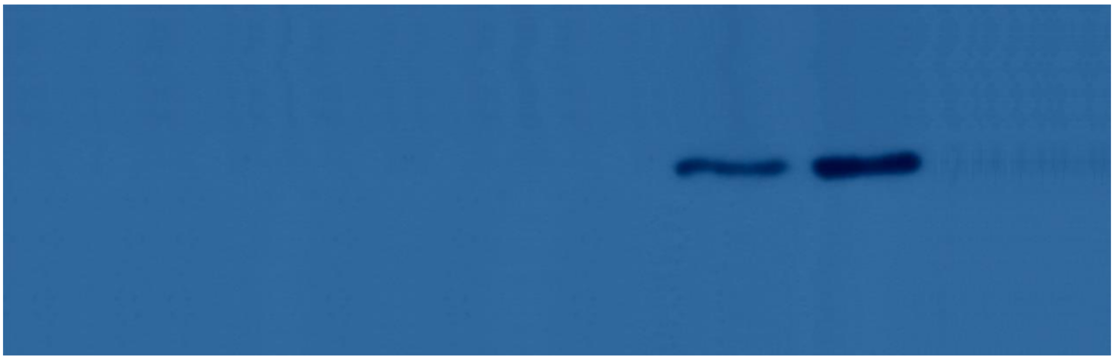

Fig.7C-2 full blot image of total CDC42

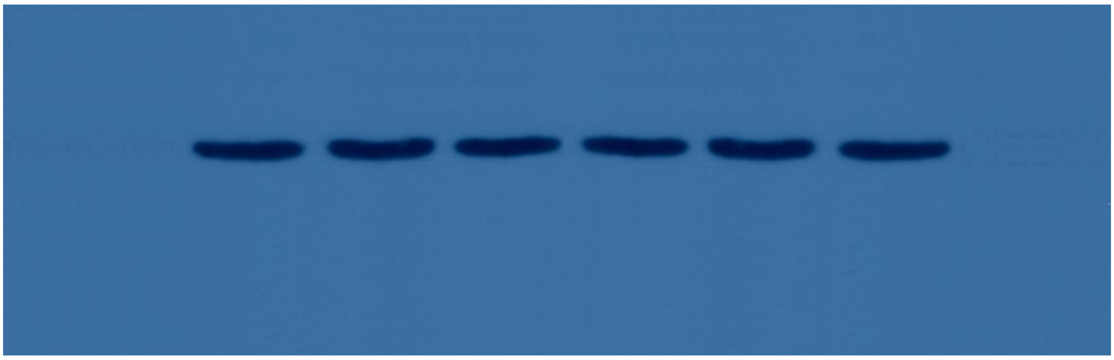

Fig.7C-3 full blot image of GTP-RAC1

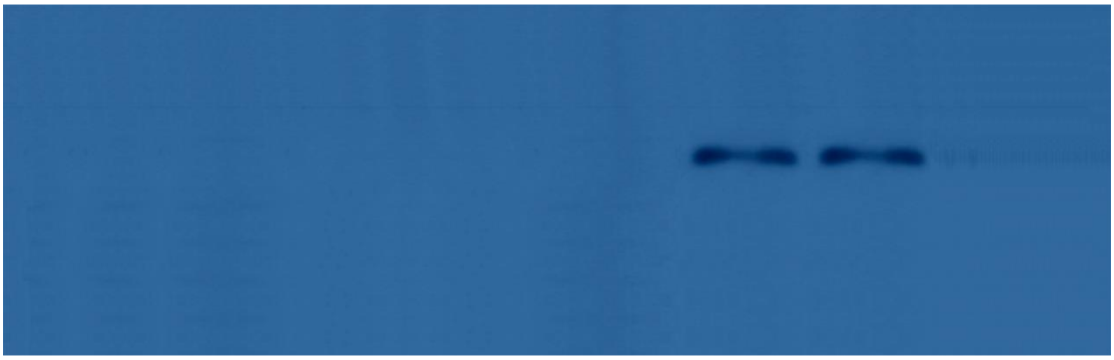

Fig.7C-4 full blot image of total RAC1

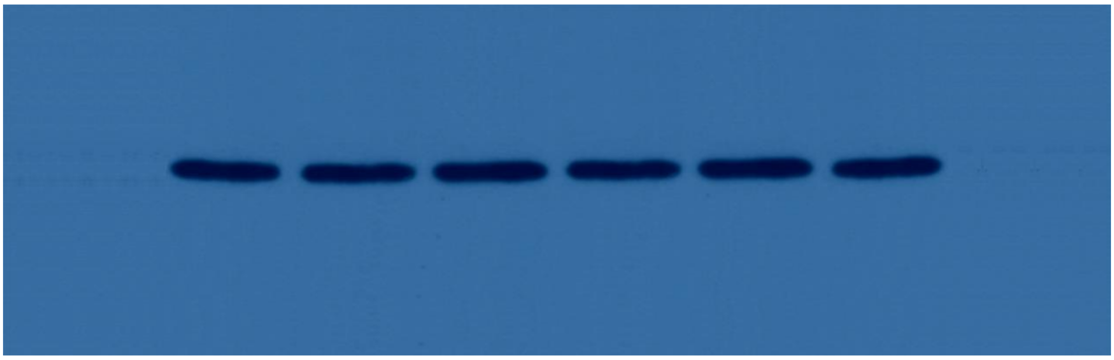

Original images for the phosphorylation levels of PAKs, RAFs and ERK1/2 in the GCs before and after the transfection with the pYr-adshuttle-4-SLIT2 construct detested by the immunoblotting. The manipulated versions are reported in Fig. 8 of the manuscript.

Fig.8A-1 full blot image of beta-actin

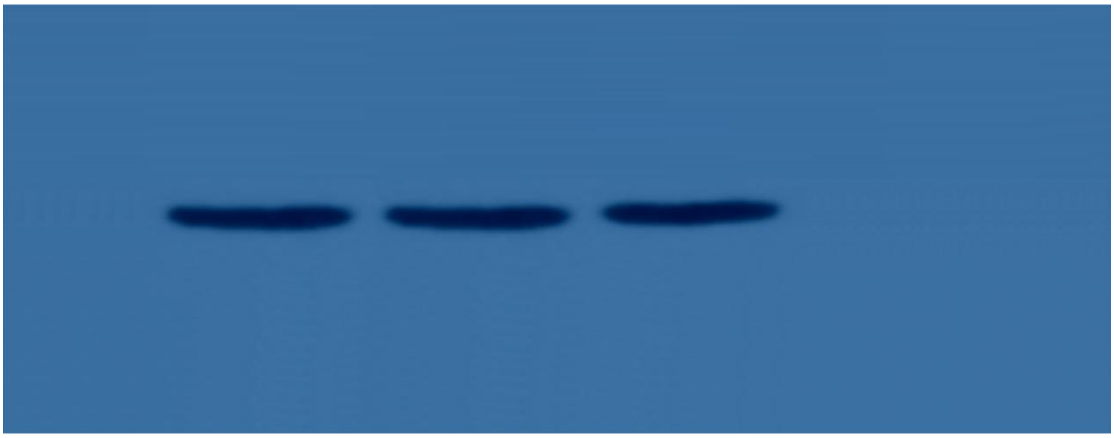

Fig.8A-2 full blot image of PAK1

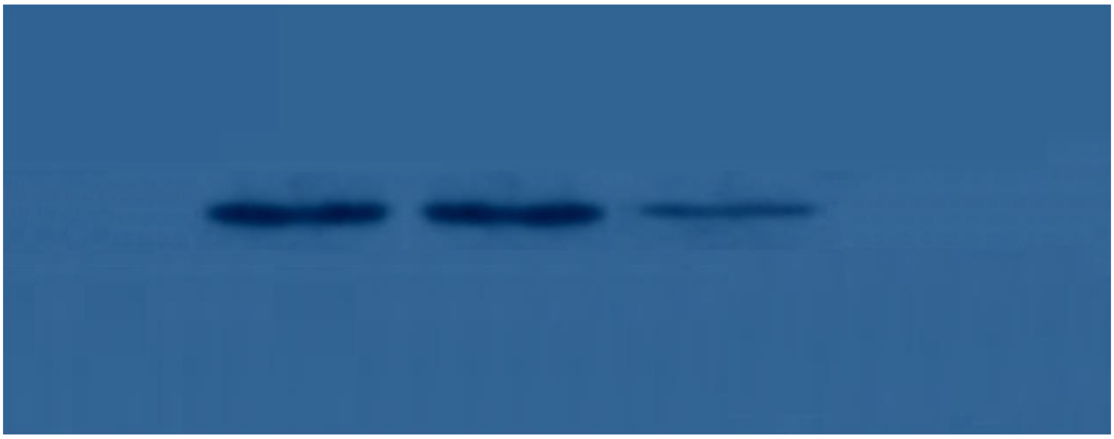

Fig.8A-3 full blot image of PAK2

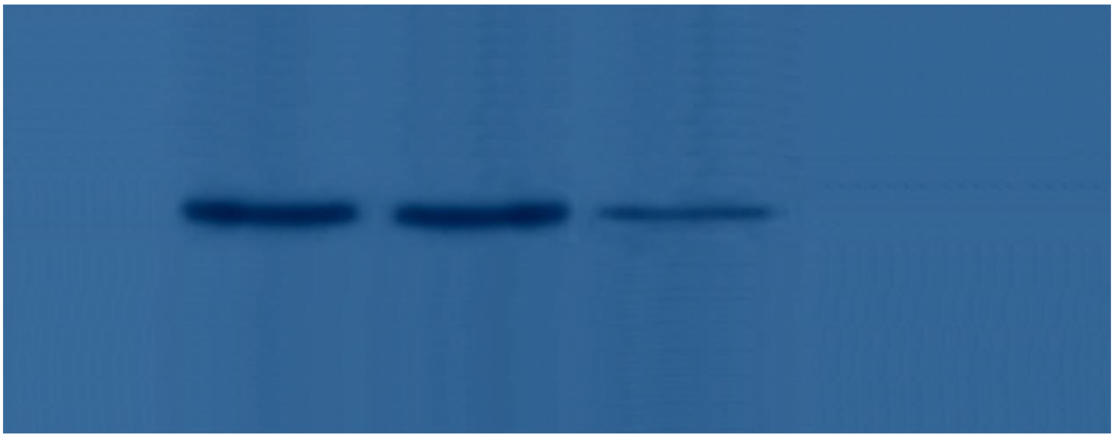

Fig.8A-4 full blot image of PAK3

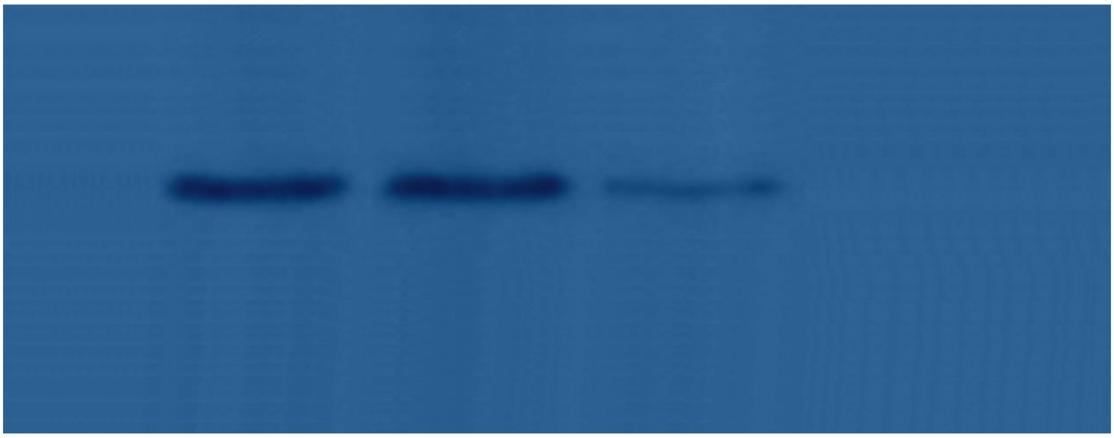

Fig.8A-5 full blot image of B-RAF

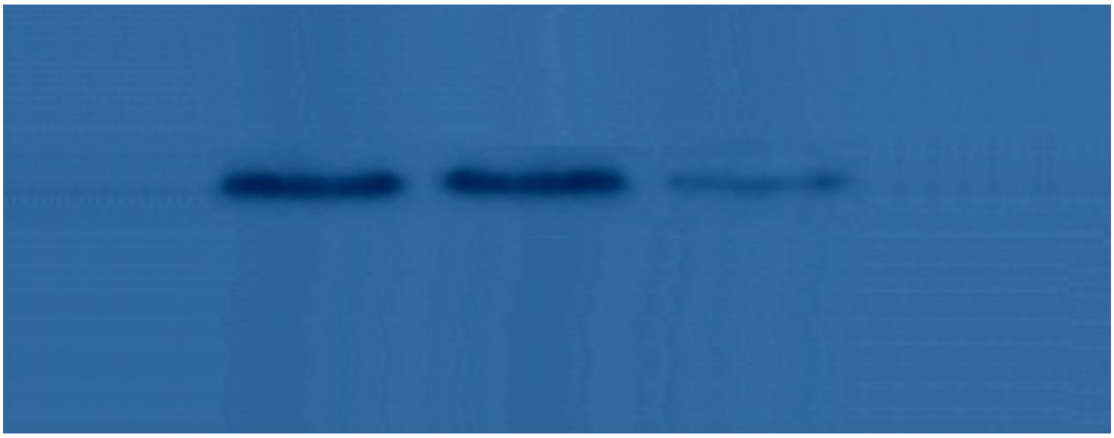

Fig.8A-6 full blot image of RAF1

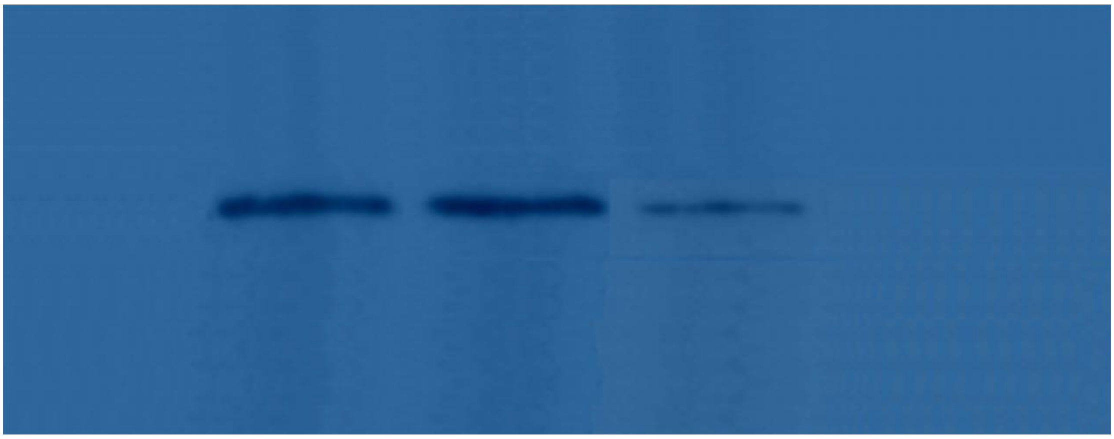

Fig.8A-7 full blot image of MEK1

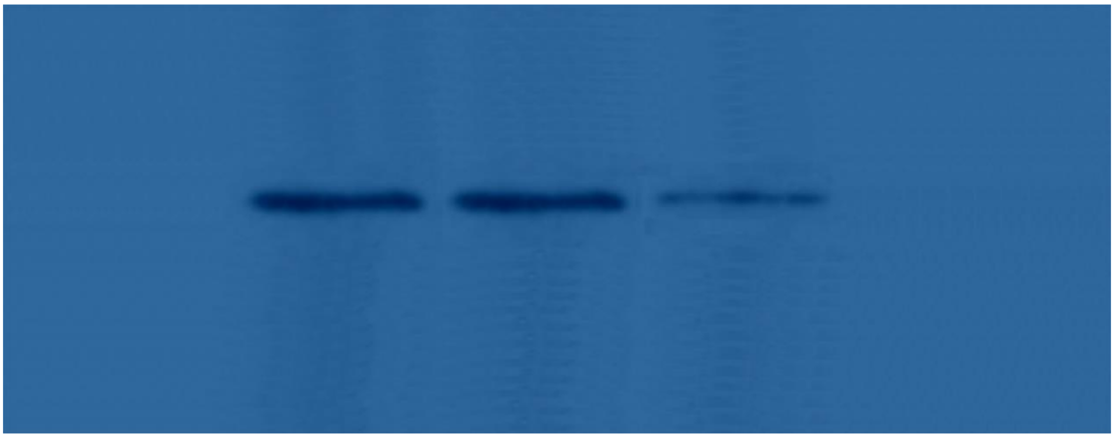

Fig.8A-8 full blot image of MEK2

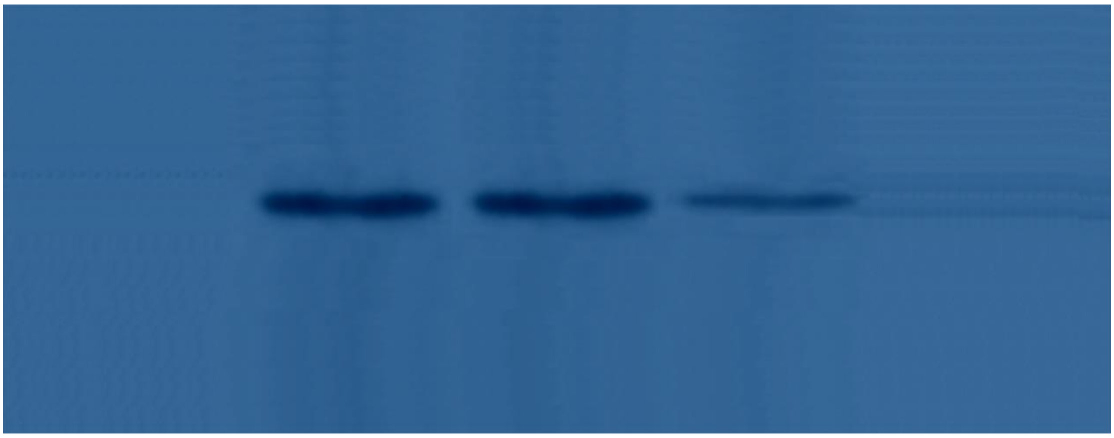

Fig.8A-9 full blot image of ERK1/2

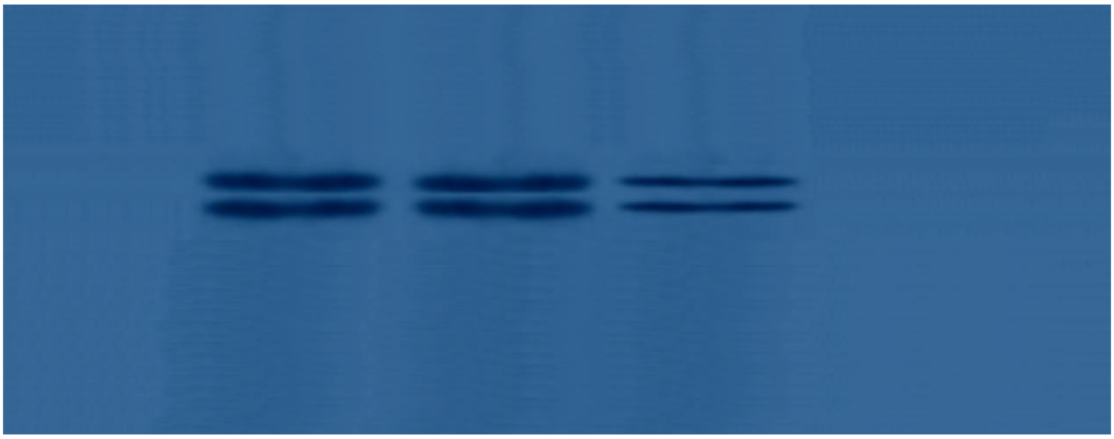

Original images for the phosphorylation levels of PAKs, RAFs and ERK1/2 in the GCs before and after the transfection with the SLIT2-specific siRNA examined by the immunoblotting. The manipulated versions are reported in Fig. 9 of the manuscript.

Fig.9A-1 full blot image of beta-actin

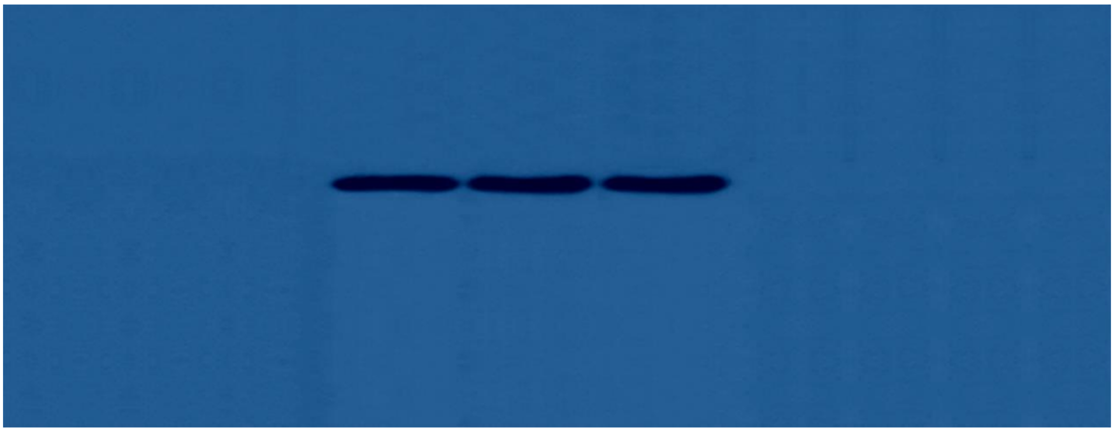

Fig.9A-2 full blot image of PAK1

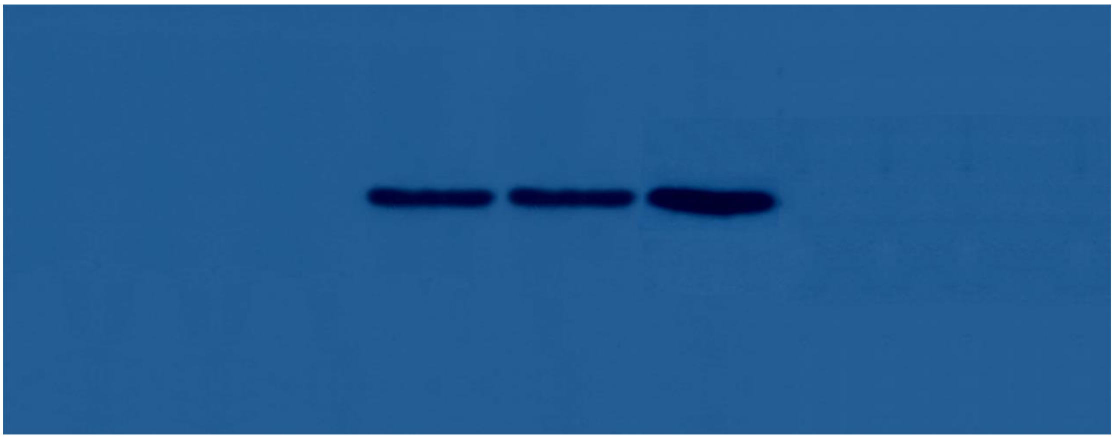

Fig.9A-3 full blot image of PAK2

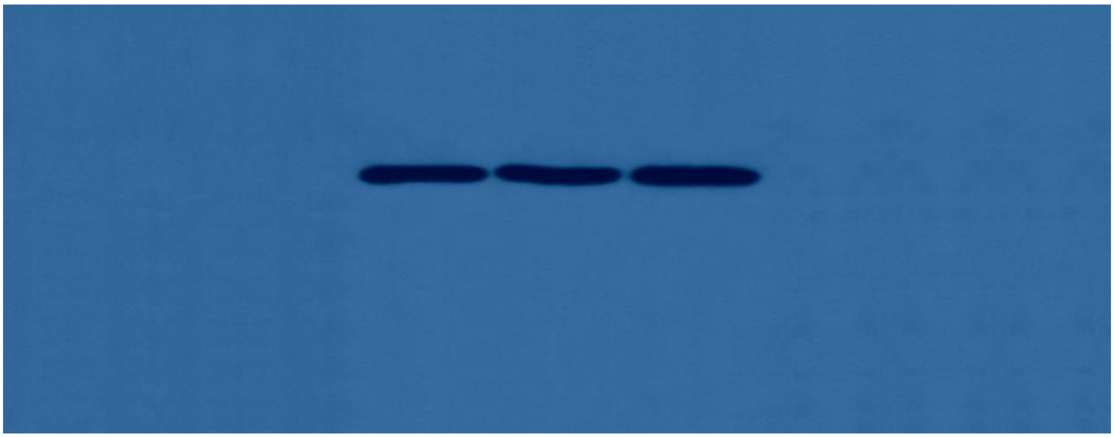

Fig.9A-4 full blot image of PAK3

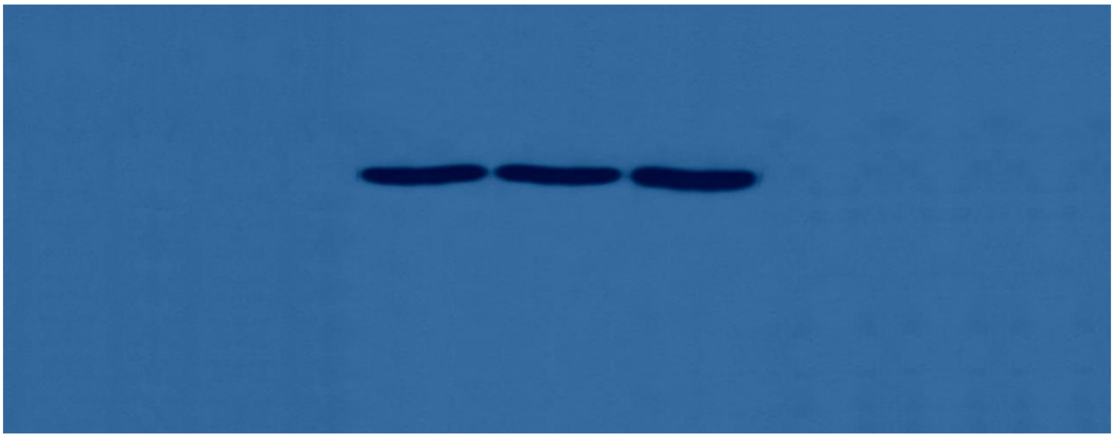

Fig.9A-5 full blot image of B-RAF

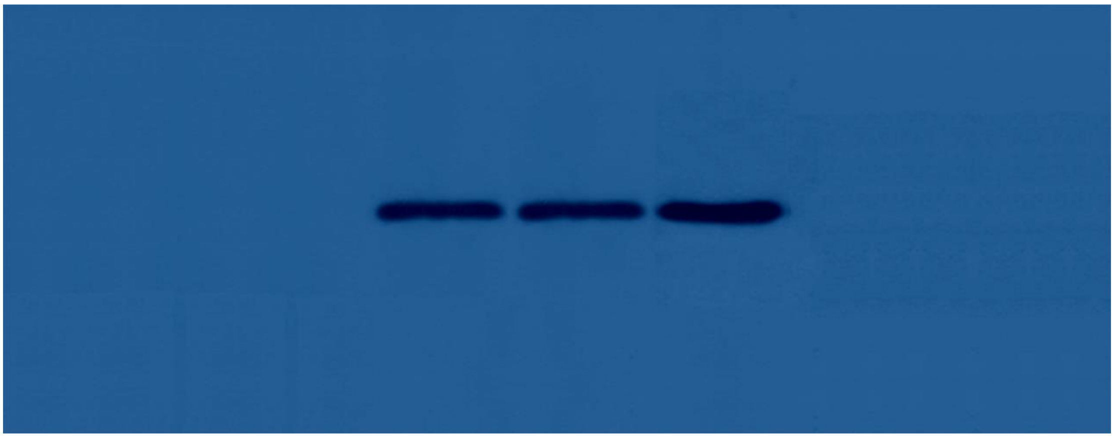

Fig.9A-6 full blot image of RAF1

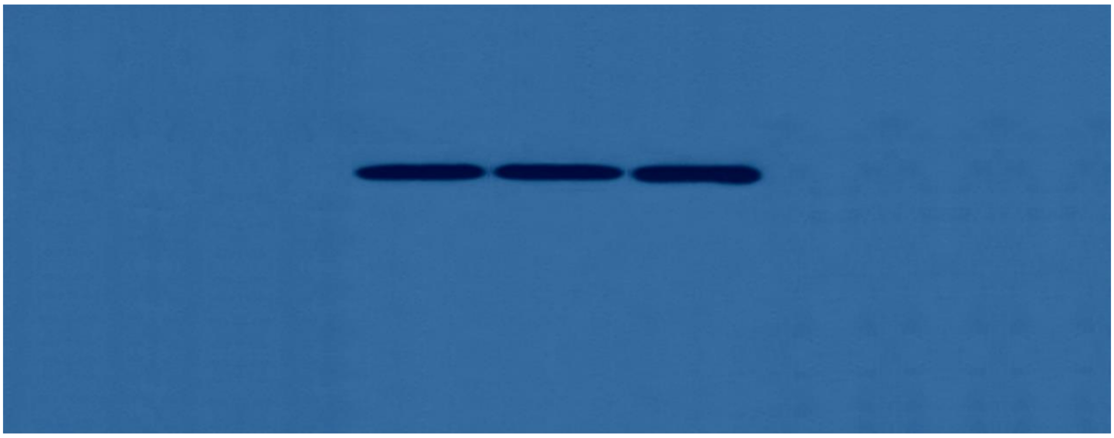

Fig.9A-7 full blot image of MEK1

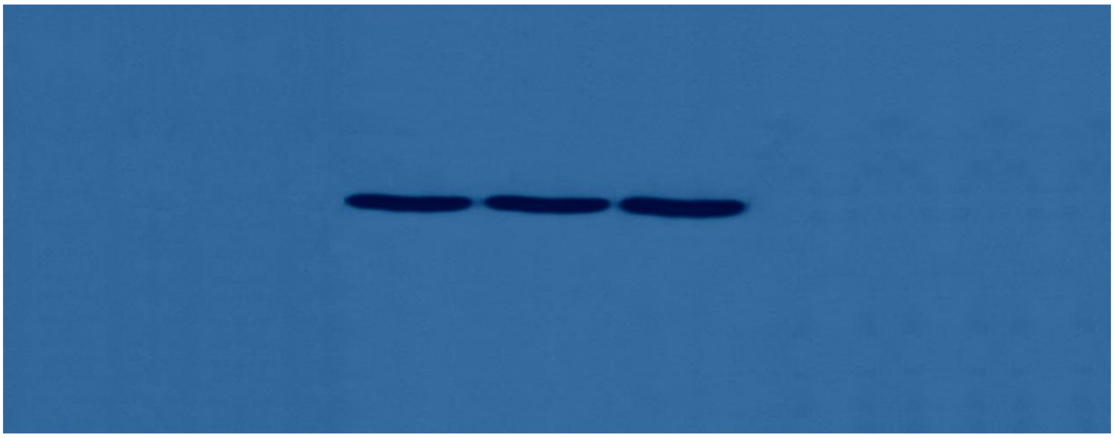

Fig.9A-8 full blot image of MEK2

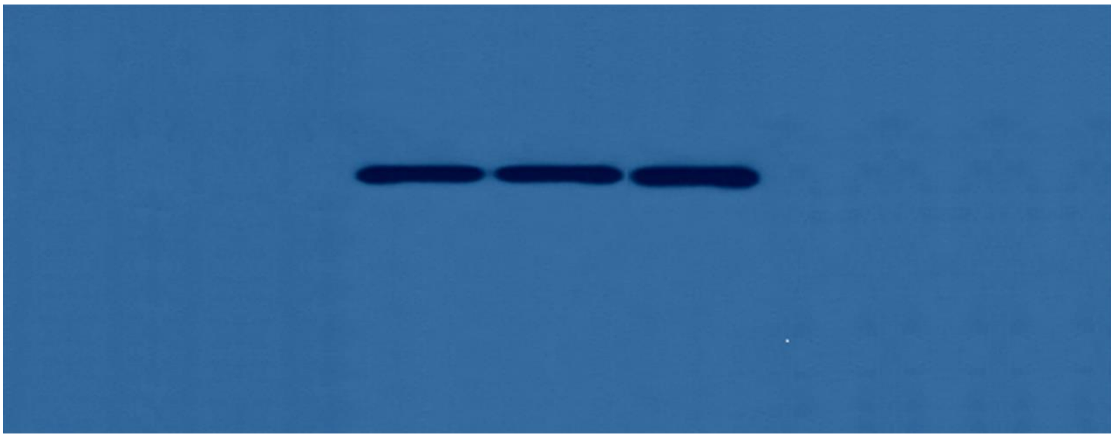

Fig.9A-9 full blot image of ERK1/2

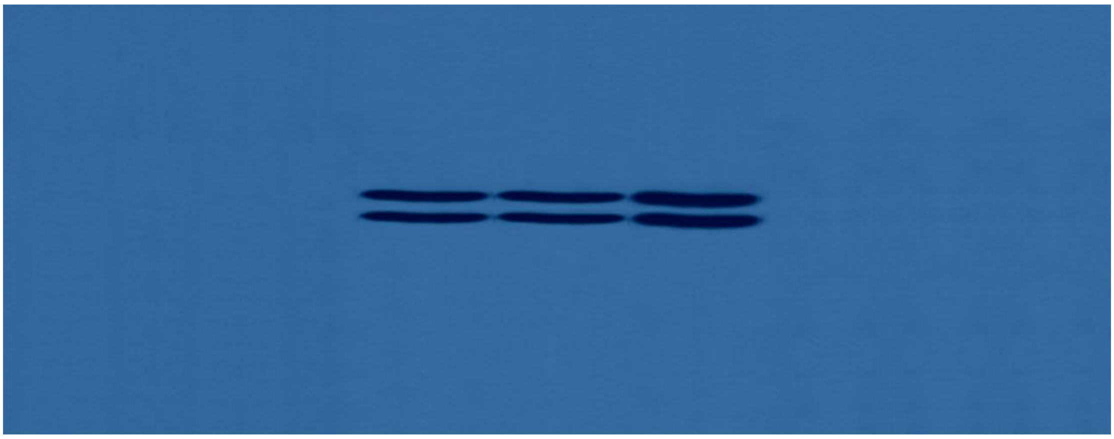

Original images for B-RAF and RAF1 expression in the GCs before and after RNAi. The manipulated versions are reported in Fig. 10 of the manuscript.

Fig. 10B-1 full blot image of B-RAF

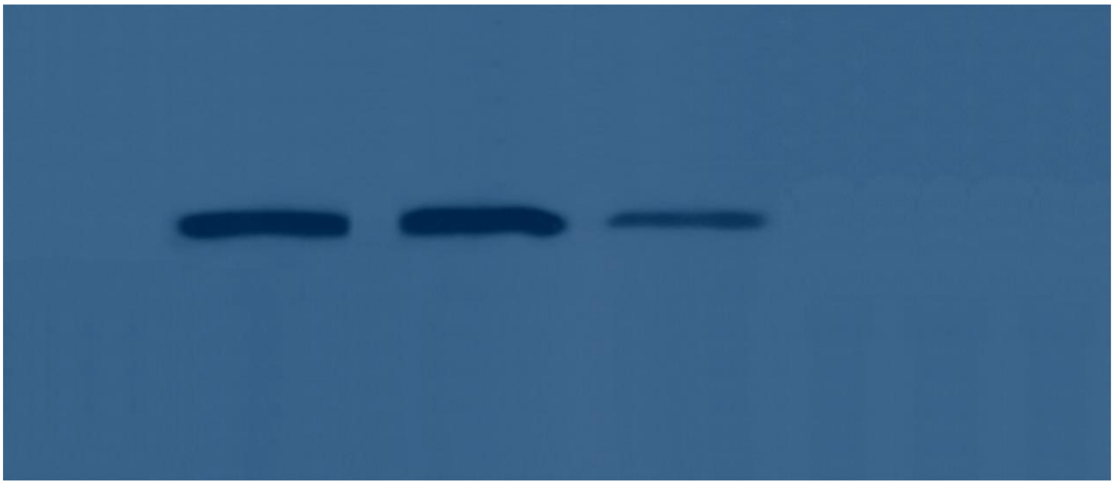

Fig. 10B-2 full blot image of RAF1

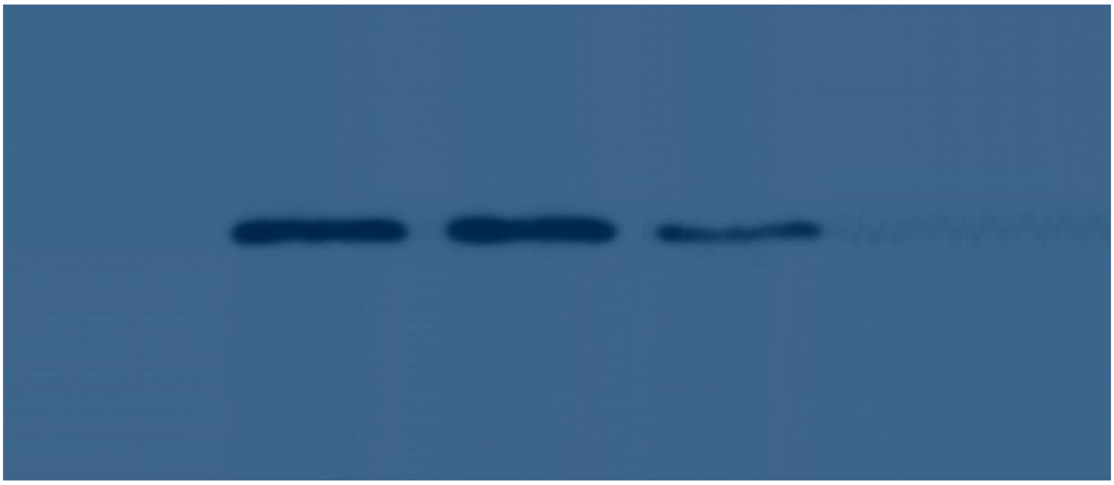

Fig. 10B-3 full blot image of beta-actin

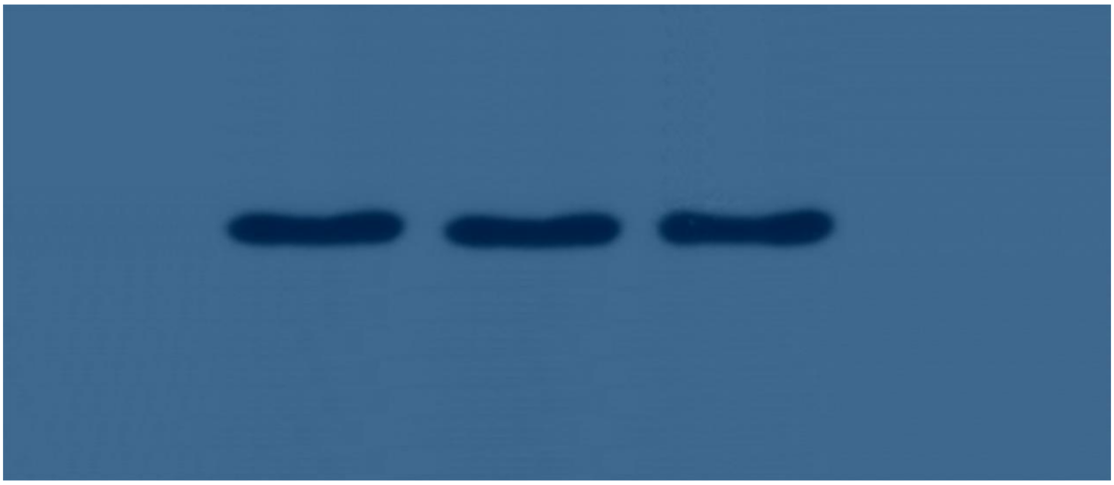

Supplement: Supplementary file 1 — Supplementary Information [file 41598_2018_27601_MOESM1_ESM.pdf]
